# Follicular Dendritic Cell-Specific Prion Protein (PrP^c^) Expression Alone Is Sufficient to Sustain Prion Infection in the Spleen

**DOI:** 10.1371/journal.ppat.1002402

**Published:** 2011-12-01

**Authors:** Laura McCulloch, Karen L. Brown, Barry M. Bradford, John Hopkins, Mick Bailey, Klaus Rajewsky, Jean C. Manson, Neil A. Mabbott

**Affiliations:** 1 The Roslin Institute & Royal (Dick) School of Veterinary Sciences, University of Edinburgh, Midlothian, United Kingdom; 2 Division of Veterinary Pathology, Infection and Immunity, School of Clinical Veterinary Science, University of Bristol, Avon, United Kingdom; 3 Program in Cellular and Molecular Medicine, Children's Hospital, and Immune Disease Institute, Harvard Medical School, Boston, Massachusetts, United States of America; Creighton University, United States of America

## Abstract

Prion diseases are characterised by the accumulation of PrP^Sc^, an abnormally folded isoform of the cellular prion protein (PrP^C^), in affected tissues. Following peripheral exposure high levels of prion-specific PrP^Sc^ accumulate first upon follicular dendritic cells (FDC) in lymphoid tissues before spreading to the CNS. Expression of PrP^C^ is mandatory for cells to sustain prion infection and FDC appear to express high levels. However, whether FDC actively replicate prions or simply acquire them from other infected cells is uncertain. In the attempts to-date to establish the role of FDC in prion pathogenesis it was not possible to dissociate the *Prnp* expression of FDC from that of the nervous system and all other non-haematopoietic lineages. This is important as FDC may simply acquire prions after synthesis by other infected cells. To establish the role of FDC in prion pathogenesis transgenic mice were created in which PrP^C^ expression was specifically “switched on” or “off” only on FDC. We show that PrP^C^-expression only on FDC is sufficient to sustain prion replication in the spleen. Furthermore, prion replication is blocked in the spleen when PrP^C^-expression is specifically ablated only on FDC. These data definitively demonstrate that FDC are the essential sites of prion replication in lymphoid tissues. The demonstration that *Prnp*-ablation only on FDC blocked splenic prion accumulation without apparent consequences for FDC status represents a novel opportunity to prevent neuroinvasion by modulation of PrP^C^ expression on FDC.

## Author Summary

Prion diseases are infectious neurological disorders and are considered to be caused by an abnormally folded infectious protein termed PrP^Sc^. Soon after infection prions accumulate first upon follicular dendritic cells (FDC) in lymphoid tissues before spreading to the brain where they cause damage to nerve cells. Cells must express the normal cellular prion protein PrP^C^ to become infected with prions. However, whether FDC are infected with prions or simply acquire them from other infected cells is unknown. To establish the role of FDC in prion disease PrP^C^ expression was specifically “switched on” or “off” only on FDC. We show that PrP^C^-expressing FDC alone are sufficient to sustain prion replication in the spleen. Furthermore, prion replication is blocked in the spleen when PrP^C^-expression is switched off only on FDC. These data definitively demonstrate that FDC are the essential sites of prion replication in lymphoid tissues.

## Introduction

Prion diseases (Transmissible spongiform encephalopathies; TSE) are sub-acute neurodegenerative diseases that affect both humans and animals. Many prion diseases, including natural sheep scrapie, bovine spongiform encephalopathy, chronic wasting disease in mule deer and elk, and kuru and variant Creutzfeldt-Jakob disease in humans, are acquired by peripheral exposure (eg: orally or via lesions to skin or mucous membranes). After peripheral exposure prions accumulate first upon follicular dendritic cells (FDC) as they make their journey from the site of infection to the CNS (a process termed, *neuroinvasion*) [1]–[7]. FDC are a unique subset of stromal cells resident within the primary B cell follicles and germinal centres of lymphoid tissues [8]. Prion accumulation upon FDC is critical for efficient disease pathogenesis as in their absence neuroinvasion are impaired [1]–[4]. From the lymphoid tissues prions invade the CNS via the peripheral nervous system [9].

During prion disease aggregations of PrP^Sc^, an abnormally folded isoform of the cellular prion protein (PrP^C^) accumulate in affected tissues. Prion infectivity co-purifies with PrP^Sc^ [10] and is considered to constitute the major, if not sole, component of infectious agent [11]. Host cells must express cellular PrP^C^ to sustain prion infection [12] and FDC appear to express high levels of PrP^C^ on the cell membrane in uninfected mice [13], [14]. Although prion neuroinvasion from peripheral sites of exposure is dependent upon the presence of FDC in lymphoid tissues, it is not known whether FDC actually replicate prions themselves. FDC characteristically trap and retain native antigen on their surfaces for long periods in the form of immune complexes, consisting of antigen-antibody and/or complement components. Prions are also considered to be acquired by FDC as complement-opsonized immune complexes [15]–[18]. Thus, during prion infection FDC might simply trap and retain PrP^Sc^-containing immune complexes on their surfaces following synthesis by other infected cells such as neurones.

Many cell types including classical DC, lymphocytes, mast cells, platelets, reticulocytes and epithelial cells secrete membrane vesicles termed exosomes that are enriched in cell-specific protein [19], [20]. Although the functions of exosomes are uncertain FDC can bind them on their surfaces. These microvesicles permit FDC to passively acquire and display proteins on their surfaces that they do not express at the mRNA level [21]. Studies have shown that prions only accumulated in the spleens of mice in which the FDC-containing stromal compartment expressed PrP^C^ [13], [14]. However, in each of those studies it was not possible to dissociate the *Prnp* expression status of the FDC from that of the nervous system and all other host-derived non-haematopoietic and stromal cell populations [13], [14], [22]. This is important as prion infection can occur within inflammatory PrP^C^-expressing stromal cells that are distinct from FDC [23]. Furthermore, as both PrP^C^ and PrP^Sc^ can be released from cells in association with exosomes [20] FDC may passively acquire PrP^C^ and prions after release in exosomes from other infected cells [24], [25].

No therapies are available to treat prions diseases. A thorough characterization of the host cells that are infected by prions is imperative for the identification of candidate molecular targets for therapeutic intervention, the development of useful pre-clinical diagnostics and to aid our understanding of the risk of transmission. To definitively determine the role of FDC in prion pathogenesis, two unique compound transgenic mouse models were created in which PrP^C^ expression was specifically “switched on” or “switched off” only on FDC. These mice were then used to establish: i) whether FDC express PrP^C^ or simply acquire it from other host cells; and ii) whether FDC amplify prions, or simply acquire them from other infected host cells. Our data clearly show that PrP^C^-expressing FDC alone are sufficient to sustain prion replication in the spleen. Furthermore, prion replication in the spleen is blocked in mice in which PrP^C^-expression is specifically ablated only on FDC.

## Results

### Mice expressing Cre recombinase specifically in FDC

**Figure 1:**
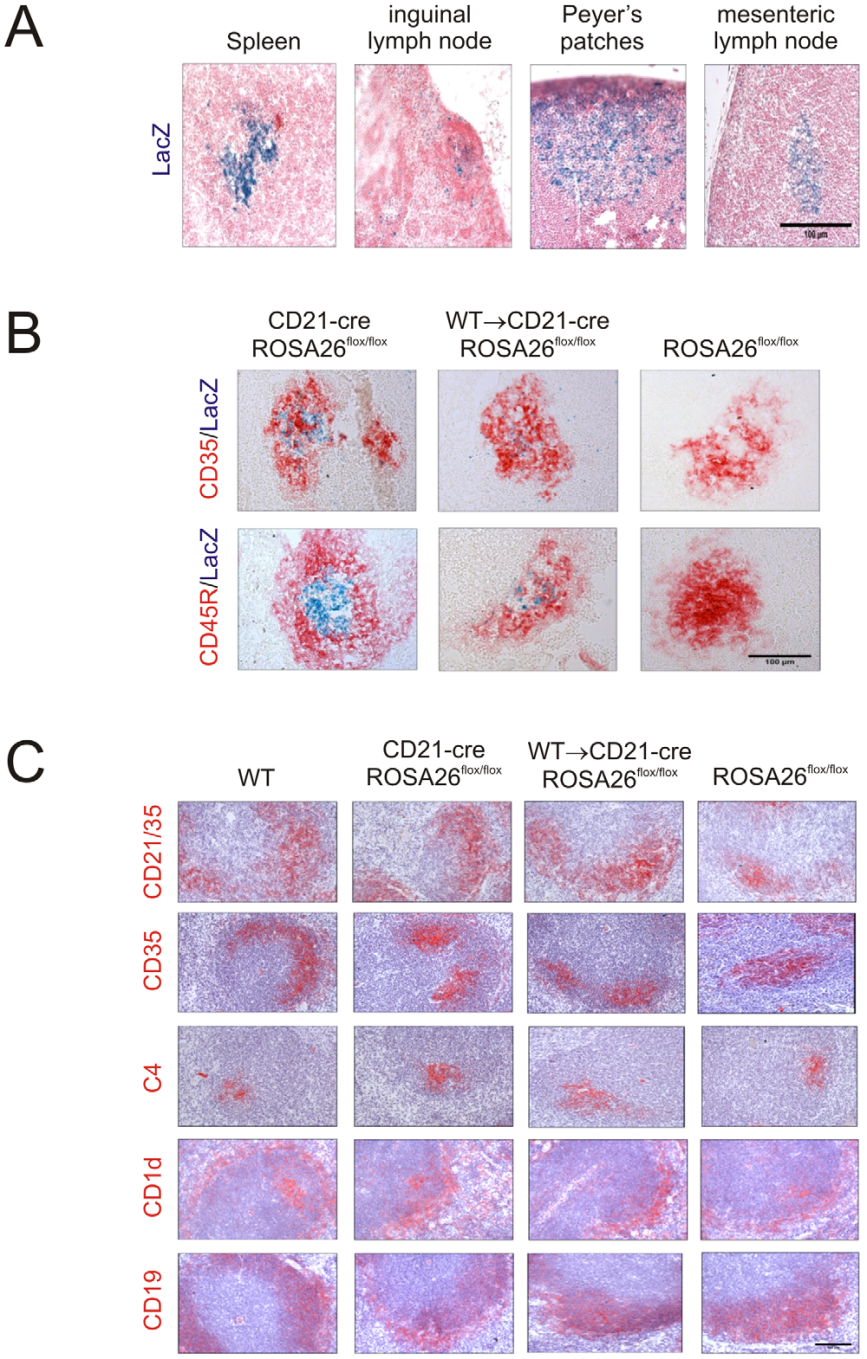
*Cre*-mediated gene recombination in FDC in the spleens, lymph nodes and Peyer's patches of CD21-Cre ROSA26^flox/Flox^ mice. A) Analysis of the cellular sites of *LacZ* expression (blue) in the spleens, inguinal lymph nodes, Peyer's patches and mesenteric lymph nodes of CD21-Cre ROSA26^flox/flox^ mice shows Cre-mediated recombination in a focus of cells within the B cell follicles. Sections were counterstained with nuclear fast red (red). B) IHC analysis of FDC (CD35^+^ cells, upper row, red) and B cells (CD45R^+^ cells, lower row, red) confirmed that Cre-mediated *LacZ* expression (blue) was associated with FDC in the spleens of WT→CD21-Cre ROSA26^flox/flox^ mice. No *LacZ* expression was associated with FDC in spleens from ROSA26^flox/flox^ mice that lacked Cre. C) IHC analysis of the status of FDC (CD35^+^ and C4-binding cells; red) and B cells expressing CD45R, CD19, and CD1d (red) in spleens from WT, CD21-Cre ROSA26^flox/flox^, WT→CD21-Cre ROSA26^flox/flox^ and ROSA26^flox/flox^ mice. Scale bars 100 µm. *n* = 6 mice/group.

To study FDC-specific gene function transgenic mice were used that expressed Cre recombinase under the control of the *Cr2* locus (CD21-Cre mice) which directs expression in FDC and mature B cells [26], [27]. First the cellular specificity of the Cre recombination was assessed by crossing the CD21-Cre mice with the ROSA26^flox/flox^ reporter strain [28]. Histological analysis showed efficient *LacZ* expression indicative of Cre-mediated gene recombination in FDC and B cell follicles in the spleens, lymph nodes and Peyer's patches of CD21-Cre ROSA26^flox/flox^ mice (Figure 1A, B). No recombination was observed in FDC and mature B cells in the spleens of ROSA26^flox/flox^ reporter mice that lacked Cre expression (Figure 1B). Unlike lymphocytes, FDC do not derive from bone marrow precursors [29]. As a consequence, it is possible to mix-and-match the genotype of FDC and lymphocytes by grafting bone marrow cells from donor mice into recipients of a different genetic background [13], [14], [22]. To restrict Cre-expression to FDC, adult CD21-Cre ROSA26^flox/flox^ mice were lethally γ-irradiated and 24 h later reconstituted with bone marrow from Cre-deficient C57BL/6 wild-type (WT) mice (termed WT→CD21-Cre ROSA26^flox/flox^ mice) and tissues from six mice from each group analysed 100 days after transfusion. Using this approach, in these mice all B cells lack Cre-expression as they derive from the WT donor bone marrow, whereas the FDC express Cre as they are host-derived. Analysis of the cellular sites of *LacZ* expression in WT→CD21-Cre ROSA26^flox/flox^ mice confirmed that Cre-mediated recombination was associated with FDC (Figure 1B). No other cellular sites of Cre-mediated recombination were observed in the spleens of WT→CD21-Cre ROSA26^flox/flox^ mice. Furthermore, no other cellular sites of Cre-mediated recombination were observed in a wide range of non-lymphoid peripheral tissues from CD21-Cre ROSA26^flox/flox^ and WT→CD21-Cre ROSA26^flox/flox^ (heart, liver, kidney, pancreas, ear, tongue, skeletal, muscle, ovary, uterus, bladder, testes, epididymis, sciatic nerve and spinal cord; data not shown). These data clearly demonstrate that CD21-Cre mice are a useful tool to study FDC-specific gene expression and function.

### Expression of Cre recombinase by the *Cr2* promoter is not toxic to FDC

Cre toxicity can occur in some Cre transgenic mouse lines whereby Cre recombinase causes mis-recombination, DNA damage and death of Cre-expressing cells [30]. However, immunohistochemical (IHC) analysis of spleens from CD21-Cre ROSA26^flox/flox^ mice and WT→CD21-Cre ROSA26^flox/flox^ mice showed no significant effect of Cre-expression on the status of FDC networks and B cell follicles when compared to spleens from WT control mice and ROSA26^flox/flox^ mice that lacked Cre expression (Figure 1C). Furthermore, the expression of Cre recombinase under the control of the *Cr2* locus had no observable effect on CD21/35 expression (Figure 1C).

### FDC express *Prnp* and do not acquire PrP^C^ from neighbouring cells

**Figure 2:**
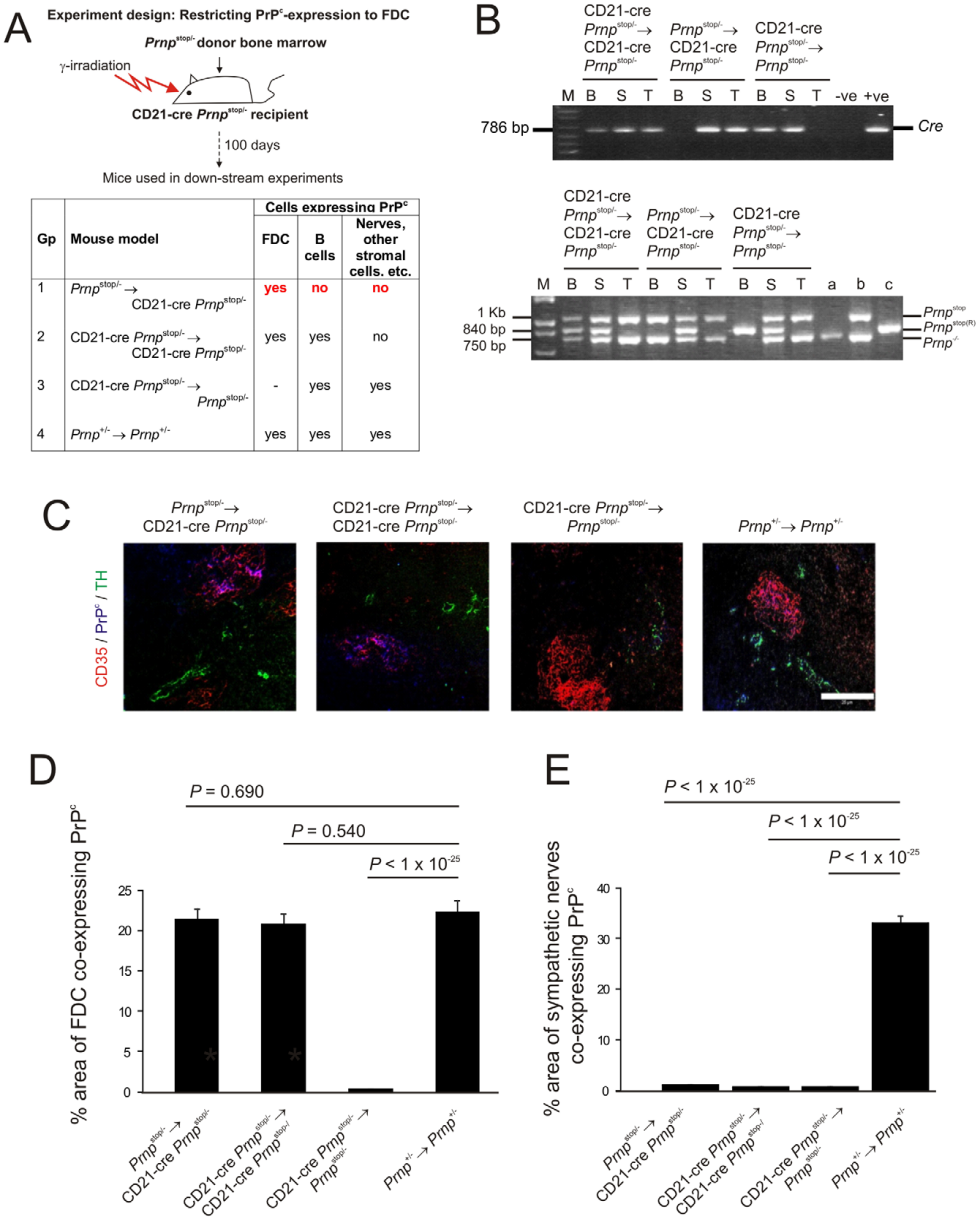
FDC-restricted PrP^c^ expression in the spleens of *Prnp* ^stop/-^→CD21-Cre *Prnp* ^stop/-^ mice. A) The anticipated distribution of PrP^C^ expression on FDC and B cells in tissues from each mouse group. B) The detection of *Cre* in the tail and spleen but not blood of the *Prnp* ^stop/-^→CD21-Cre *Prnp* ^stop/-^ mice confirmed the restriction of the *Cre*-expression to the stromal but not haematopoietic compartments of these mice (upper panel). Efficient Cre-mediated recombination of *Prnp^stop^* (*Prnp^stop^* ^(R)^) was restricted to the FDC-containing stromal compartment of the spleens of *Prnp* ^stop/-^→CD21-Cre *Prnp* ^stop/-^ mice when compared to control mice. Cre-mediated recombination by CD21-expressing lymphocytes was efficiently prevented in these mice by the irradiation and transfer of *Prnp* ^stop/-^ bone marrow as demonstrated by the lack of a *Prnp* ^stop(R)^ band in DNA extracted from blood (lower panel). B, blood; S, spleen; T, tail; M, DNA size markers; a, b, c, control DNA samples for each transgene combination tested which were (a) *Prnp* ^-/-^, (b) *Prnp* ^stop/-^ and (c) complete recombination of the stop cassette within the *Prnp* ^stop/-^ allele. C) IHC analysis of PrP^C^ expression (blue) by FDC (CD35^+^ cells; red) and sympathetic nerves (TH^+^ cells, green) confirmed PrP^C^ expression was restricted to FDC in spleens of *Prnp* ^stop/-^→CD21-Cre *Prnp* ^stop/-^ mice. Scale bar, 100 µm. D) Morphometric analysis confirmed that the magnitude of the PrP^C^ expression co-localized upon the surfaces of FDC in the spleens of *Prnp* ^stop/-^→CD21-Cre *Prnp* ^stop/-^ mice was similar to that observed upon FDC in spleens from *Prnp* ^+/-^→*Prnp* ^+/-^ control mice (*p*<0.690, *n* = 48 FDC networks/group). In contrast, in the absence of Cre-recombinase expression by FDC in CD21-Cre *Prnp* ^stop/-^→*Prnp* ^stop/-^ mice, PrP^C^ expression was substantially lower than that observed upon FDC in spleens from *Prnp* ^+/-^→*Prnp* ^+/-^ control mice (*p*<1×10^-25^, *n* = 48 FDC networks/group). E) Morphometric analysis confirmed that PrP^C^ expression upon the surfaces of sympathetic nerves in the spleens of *Prnp* ^stop/-^→CD21-Cre *Prnp* ^stop/-^, CD21-Cre *Prnp* ^stop/-^→ CD21-Cre *Prnp* ^stop/-^ and CD21-Cre *Prnp* ^stop/-^→*Prnp* ^stop/-^ mice was significantly ablated when compared to that observed upon sympathetic nerves in spleens from *Prnp* ^+/-^→*Prnp* ^+/-^ control mice (*p*<1×10^-25^, *n* = 48 sympathetic nerves/group). For all panels *n* = 6 mice/group.

Next, mice were created in which *Prnp* expression (which encodes PrP^C^) was restricted only to FDC. To do so, CD21-Cre mice were first bred onto a PrP^C^-deficient (*Prnp* ^-/-^) background. The resulting CD21-Cre *Prnp* ^-/-^ mice were then crossed with *Prnp* ^stop/-^ mice in which a floxed β-geo stop cassette was inserted into intron 2 of the *Prnp* gene upstream of exon 3 [31]. In the progeny CD21-Cre *Prnp* ^stop/-^ mice, PrP^C^ is only expressed in cells expressing Cre recombinase (CD21-expressing FDC and mature B cells). To restrict the *Prnp*-expression to FDC, CD21-Cre *Prnp* ^stop/-^ mice were lethally γ-irradiated and grafted with bone marrow from Cre-deficient *Prnp* ^stop/-^ mice (*Prnp* ^stop/-^→CD21-Cre *Prnp* ^stop/-^ mice). We also performed bone marrow transfers from CD21-Cre *Prnp* ^stop/-^ donors into CD21-Cre *Prnp* ^stop/-^ recipients (CD21-Cre *Prnp* ^stop/-^→CD21-Cre *Prnp* ^stop/-^ mice), CD21-Cre *Prnp* ^stop/-^ donors into Cre-deficient *Prnp* ^stop/-^ mice (CD21-Cre *Prnp* ^stop/-^→*Prnp* ^stop/-^ mice) and *Prnp* ^+/-^ donors into *Prnp* ^+/-^ recipients (*Prnp* ^+/-^→*Prnp* ^+/-^ mice) as controls (Figure 2A).

Spleens, tails and blood from six mice from each group were examined 100 days after bone marrow transfusion. PCR analysis of DNA isolated from the tails, blood and spleens of mice in each group was used to confirm the presence of *Cre* (Figure 2B, upper panel) and Cre-mediated DNA recombination (Figure 2B, lower panel) within the stromal, haematopoietic or both compartments (respectively). The detection of *Cre* in the tail and spleen but not blood of the *Prnp* ^stop/-^→CD21-Cre *Prnp* ^stop/-^ mice confirmed the restriction of the *Cre*-expression to the stromal but not haematopoietic compartments of these mice. In addition, PCR analysis also confirmed that in these mice efficient Cre-mediated recombination of the *Prnp* ^stop/-^ allele was restricted to the FDC-containing stromal compartment of the spleen (Figure 2B). In *Prnp* ^stop/-^→CD21-Cre *Prnp* ^stop/-^ mice the recombined *Prnp* ^stop/-^ allele (*Prnp* ^stop(R)^) was detected in the spleen, but not blood and tail. Thus these data indicate that in the spleens of *Prnp* ^stop/-^→CD21-Cre *Prnp* ^stop/-^ mice Cre-mediated recombination is restricted to FDC and not B cells.

As anticipated, in the spleens of *Prnp* ^+/-^→*Prnp* ^+/-^ control mice high levels of PrP^C^ expression were observed upon FDC and tyrosine hydroxylase (TH)-positive sympathetic nerves (Figure 2C). In contrast, in the spleens of *Prnp* ^stop/-^→CD21-Cre *Prnp* ^stop/-^ mice PrP^C^ was only expressed on FDC (Figure 2C). In the absence of Cre-recombinase expression by FDC and peripheral nerves in CD21-Cre *Prnp* ^stop/-^→*Prnp* ^stop/-^ mice, PrP^C^ expression was not expressed by either cell population (Figure 2C).

Morphometric analysis confirmed that the amount of the PrP^C^ expression co-localized upon the surfaces of FDC in the spleens of *Prnp* ^stop/-^→CD21-Cre *Prnp* ^stop/-^ mice was not significantly different from that observed upon FDC in spleens from *Prnp* ^+/-^→*Prnp* ^+/-^ control mice (*P*<0.69, *n* = 48 FDC/group; Figure 2D). In contrast, in the absence of Cre-recombinase expression by FDC in CD21-Cre *Prnp* ^stop/-^→*Prnp* ^stop/-^ mice, PrP^C^ expression was substantially lower than that observed upon FDC in spleens from *Prnp* ^+/-^→*Prnp* ^+/-^ control mice (*P*<1×10^-25^, *n* = 48; Figure 2D). Morphometric analysis also confirmed that PrP^C^ expression upon the surfaces of sympathetic nerves in the spleens of *Prnp* ^stop/-^→CD21-Cre *Prnp* ^stop/-^, CD21-Cre *Prnp* ^stop/-^→ CD21-Cre *Prnp* ^stop/-^ and CD21-Cre *Prnp* ^stop/-^→*Prnp* ^stop/-^ mice was significantly ablated when compared to that observed upon sympathetic nerves in spleens from *Prnp* ^+/-^→*Prnp* ^+/-^ control mice (*p*<1×10^−25^, *n* = 48 sympathetic nerves/group). Together, these data confirm that in the spleens of *Prnp* ^stop/-^→CD21-Cre *Prnp* ^stop/-^ mice PrP^C^ expression is specifically restricted to FDC, whereas in spleens from CD21-Cre *Prnp* ^stop/-^→*Prnp* ^stop/-^ mice, FDC lack PrP^C^ expression. FDC can passively acquire the expression of some surface molecules including MHC class II and complement component C4 [21], [32]. However, these data confirm that FDC express high levels of cellular PrP^C^ on their surfaces and do not simply acquire it from neighbouring cells.

**Figure 3:**
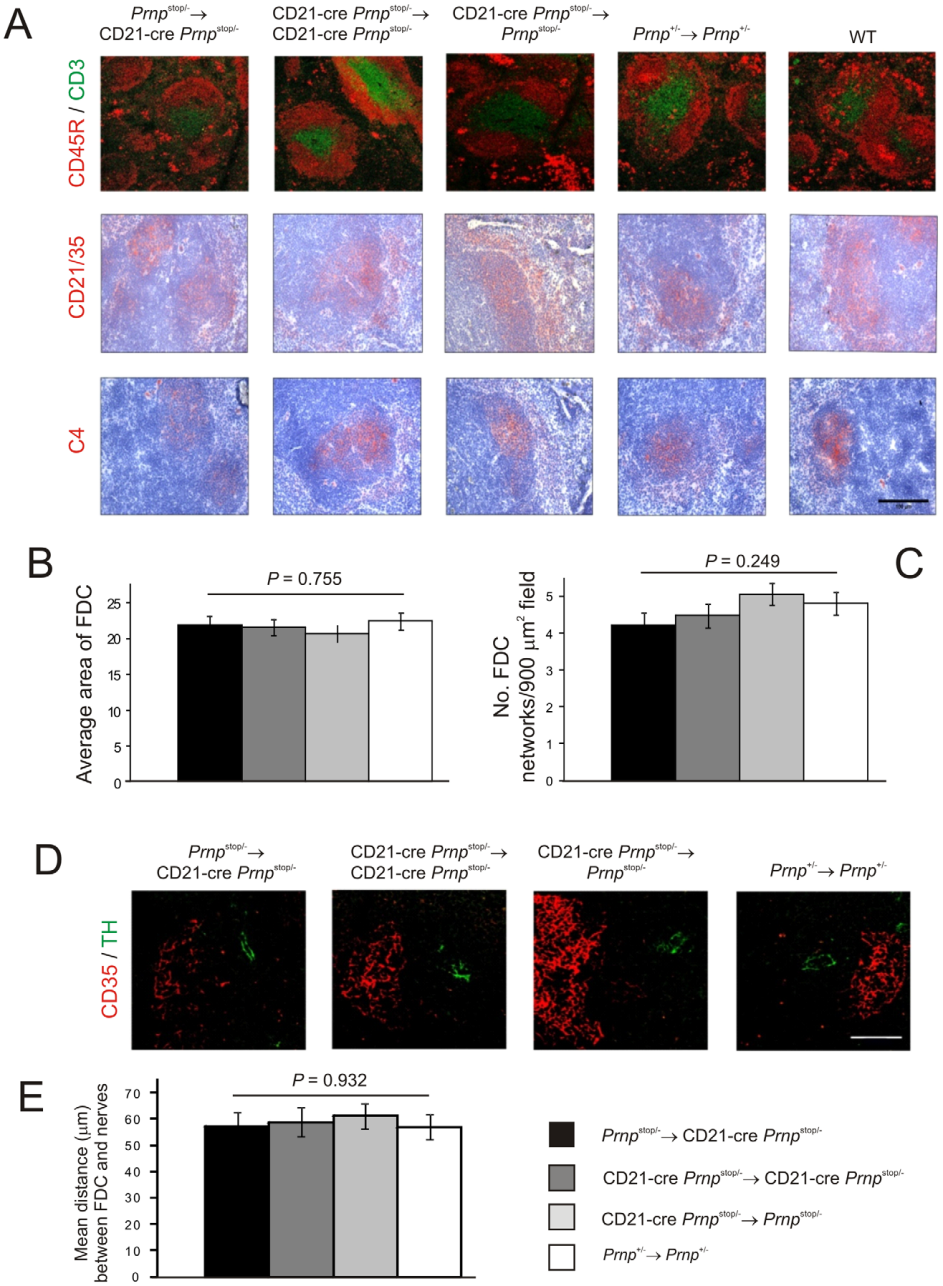
Effect of FDC-restricted PrP^c^ expression on the spleens of *Prnp* ^stop/-^→CD21-Cre *Prnp* ^stop/-^ mice. A) IHC analysis of the status of FDC networks (C4-binding cells and CD21/CD35^+^ cells; red), B cells expressing CD45R (red), and CD3^+^ T cells (green). Morphometric analysis confirmed that there were no significant difference in the size (B) and number (C) of the CD35^+^ FDC networks in spleens from *Prnp* ^stop/-^→CD21-Cre *Prnp* ^stop/-^, CD21-Cre *Prnp* ^stop/-^→CD21-Cre *Prnp* ^stop/-^, CD21-Cre *Prnp* ^stop/-^→*Prnp* ^stop/-^ mice and *Prnp* ^+/-^→*Prnp* ^+/-^ control mice (*n* = 32 FDC networks/group). D and E), Comparison of the sympathetic innervation in spleens from each mouse group. D) IHC detection of TH-positive sympathetic nerves (green) and FDCs (CD35^+^ cells; red). Scale bar, 50 µm. E) Quantitative analysis of the relative positioning of the FDC and sympathetic nerves showed there were no significant differences in average distance between these cell populations in spleens from each mouse group (*P* = 0.932, *n* = 48 FDC networks/group). For all panels *n* = 6 mice/group.

IHC analysis confirmed that the microarchitecture (Figure 3A), size (*P = 0.*755, *n* = 32; Figure 3B) and number (*P = 0.*249, *n* = 32; Figure 3C) of the FDC networks in spleens from mice with *Prnp*-expression restricted to FDC (*Prnp* ^stop/-^→CD21-Cre *Prnp* ^stop/-^ mice) were normal when compared to control mice. Other studies have shown that the density of sympathetic nerves can significantly influence the amount of prion accumulation within in the spleen [33]. Quantitative analysis of the relative positioning of FDC and sympathetic nerves showed there were no significant differences in average distance between these cell populations in spleens from each mouse group (Figure 3D & E; *P*<0.932, *n* = 48).

### FDC-restricted PrP^C^-expression is sufficient to sustain prion replication in the spleen

Next, we determined the effect of FDC-restricted *Prnp*-expression on prion replication in the spleen. In this study, the normal cellular form of the prion protein is referred to as PrP^C^, and two distinct terms (PrP^Sc^ or PrP^d^) are used to describe the disease-specific, abnormal accumulations of PrP that are characteristically found only in prion-affected tissues and considered a reliable biochemical marker for the presence of infectious prions [10]. Disease-specific PrP (PrP^d^) accumulations are relatively resistant to proteinase K (PK) digestion, whereas cellular PrP^C^ is destroyed. Where we were able to confirm this resistance by treatment of samples with PK and subsequent paraffin-embedded tissue (PET) immunoblot analysis [34], PrP^Sc^ is used as a biochemical marker for the presence of prions. Unfortunately, treatment of tissue sections with PK destroys the microarchitecture. Therefore, for IHC analysis tissue sections were fixed and pre-treated to enhance the detection of the disease-specific abnormal accumulations of PrP (PrP^d^), whereas cellular PrP^C^ is denatured by these treatments [4]. We have repeatedly shown in a series of studies that these PrP^d^-accumulations occur only in prion-infected tissues, and correlate closely with the presence of ME7 scrapie prions [1], [4], [13], [35]–[37].

**Figure 4:**
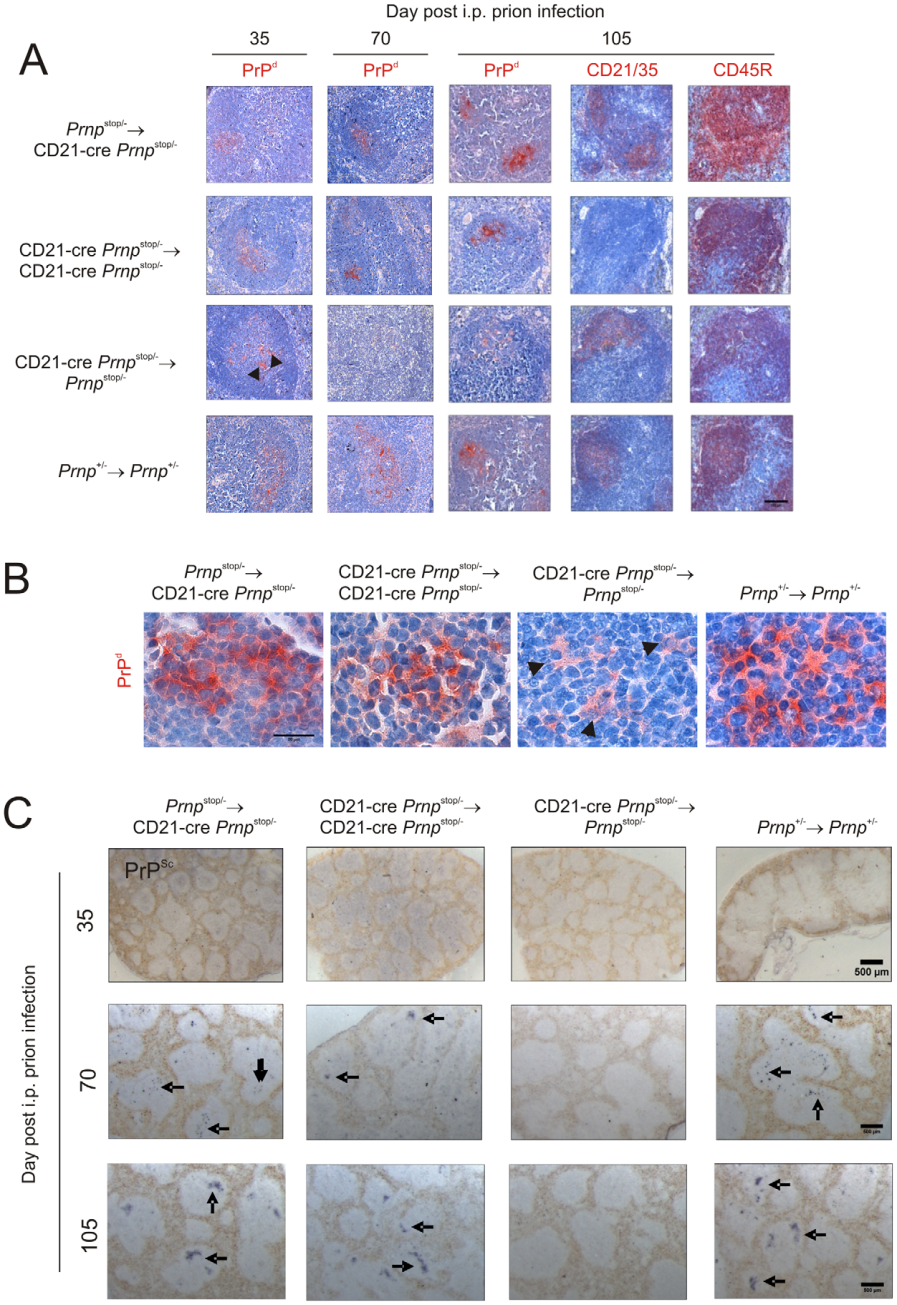
Effect of FDC-restricted PrP^c^ expression on PrP^Sc^ accumulation in the spleen. Mice were injected i.p. with the ME7 scrapie agent and tissues collected 35, 70 days and 105 days after exposure. A and B) High levels of PrP^d^ were detected in association with FDC (CD21/35 positive cells) in the B cell follicles (CD45R positive cells) of spleens of mice with PrP^C^-expressing FDC: *Prnp* ^stop/-^→CD21-Cre *Prnp* ^stop/-^ mice, CD21-Cre *Prnp* ^stop/-^→CD21-Cre *Prnp* ^stop/-^ mice and *Prnp* ^+/-^→*Prnp* ^+/-^ control mice. B) High magnification images of the sites of PrP^d^ accumulation (red) at 70 days post-injection with scrapie. Arrowheads show PrP-accumulation within tingible body macrophages. C) Analysis of adjacent sections by PET-immunoblot analysis confirmed the presence of PK-resistant PrP^Sc^ (blue/black). In contrast, no PrP^d^ or PrP^Sc^ was detected in spleens of CD21-Cre→*Prnp* ^stop/-^mice that lacked PrP^C^-expressing FDC. Arrows indicate PrP^Sc^ accumulation upon FDC. A, scale bar  = 100 µm. B, scale bar  = 20 µm. C, scale bar  = 500 µm. For all panels *n* = 4 mice/group.

Within weeks after i.p. exposure of WT mice to ME7 scrapie prions, strong accumulations of prion-specific PrP^Sc^ occur upon FDCs within the spleen and are sustained until the terminal stages of disease [1], [13], [35]. Here, mice were injected i.p. with ME7 scrapie prions and spleens from 4 mice from each group collected 35, 70 and 105 days after exposure. In spleens from control mice (*Prnp* ^+/-^→*Prnp* ^+/-^ mice) heavy PrP^d^ accumulations, consistent with localisation upon FDC, were detected at 70 days after i.p. injection with the scrapie agent and had increased in intensity by 105 days after infection (Figure 4A & B). PET immunoblot confirmed the presence of PrP^Sc^ upon the surfaces of the FDC in spleens from control mice (Figure 4C). Furthermore, in the spleens of *Prnp* ^stop/-^→CD21-Cre *Prnp* ^stop/-^ mice in which cellular PrP^C^ was expressed only on FDC, heavy PrP^Sc^ accumulations were likewise maintained upon FDC (Figure 4A & B). In contrast, in the absence of PrP^C^ expression by FDC in the spleens of CD21-Cre *Prnp* ^stop/-^→*Prnp* ^stop/-^ mice, no PrP^Sc^ accumulations were observed upon FDC. In the spleens of mice with PrP^C^-deficient FDC, if PrP was detected at all, it was only occasionally observed within tingible body macrophages (Figure 4A and B, arrowheads; Figure S1). We also analysed prion infectivity levels in spleens collected 70 days after infection from control mice (*Prnp* ^+/-^→*Prnp* ^+/-^ mice) and *Prnp* ^stop/-^→CD21-Cre *Prnp* ^stop/-^ mice in which cellular PrP^C^ was expressed only on FDC (Figure S2; *n* = 3/group). As anticipated high levels of prion infectivity were observed in each control spleen. Furthermore, consistent with data above our analysis showed that PrP^c^ expression only of FDC was sufficient to sustain high levels of prion infectivity within the spleen (Figure S2). These data demonstrate that PrP^C^ expression only on FDC is sufficient to sustain prion replication in the spleen. In the absence of PrP^C^ expression on FDC the prions appeared to be scavenged by tingible body macrophages resident within the B cell follicles.

### FDC-specific *Prnp*-ablation

**Figure 5:**
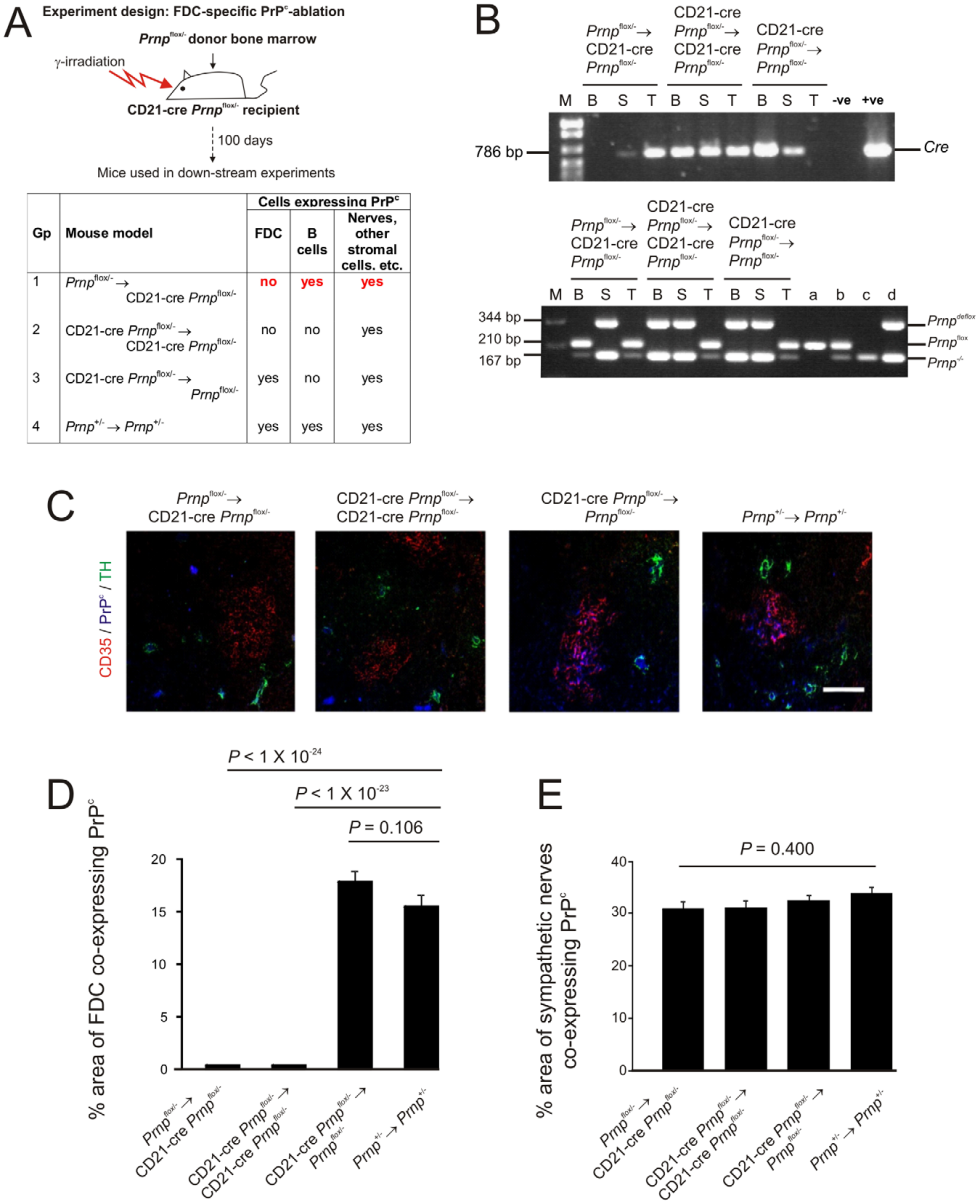
FDC-restricted PrP^c^-ablation in the spleens of *Prnp* ^flox/-^→CD21-Cre *Prnp* ^flox/-^ mice. A) The anticipated distribution of PrP^C^ expression on FDC and B cells in tissues from each mouse group. B) PCR analysis of DNA isolated from the spleens, blood and tails of *Prnp* ^flox/-^→CD21-Cre *Prnp* ^flox/-^ mice confirmed that efficient Cre-mediated DNA recombination and *Prnp*-ablation (*Prnp* ^deflox^) was restricted to the FDC-containing stromal compartment of the spleen. Cre-mediated recombination of CD21-expressing lymphocytes was efficiently prevented in these mice by the irradiation and transfer of *Prnp* ^flox/-^ bone marrow as demonstrated by the lack of a *Prnp* ^deflox^ band in DNA extracted from blood (lower panel). B, blood; S, spleen; T, tail; M, DNA size markers; a, b, c, d control DNA samples for each transgene combination tested which were (a) *Prnp* ^flox/flox^,(b) *Prnp* ^flox/-^, (c) *Prnp* ^-/-^ and (d) *Prnp* ^flox/-^ with complete recombination of the floxed exon 3. C) IHC analysis of PrP^C^ expression (blue) by FDC (CD35^+^ cells; red) and sympathetic nerves (TH^+^ cells, green) confirmed the PrP^C^-ablation was restricted to FDC in spleens of *Prnp* ^flox/-^→CD21-Cre *Prnp* ^flox/-^ mice. Scale bar, 100 µm. D) Morphometric analysis confirmed that the magnitude of the PrP^C^ expression co-localized upon the surfaces of FDC in the spleens of *Prnp* ^flox/-^→CD21-Cre *Prnp* ^flox/-^ mice was significantly lower than that observed upon FDC from *Prnp* ^+/-^→*Prnp* ^+/-^ control mice (*P*<1.0×10^-24^, *n* = 48 FDC networks/group). In contrast, in the absence of Cre-recombinase expression by FDC in CD21-Cre *Prnp* ^flox/-^→*Prnp* ^flox/-^mice, PrP^C^ expression was similar to that observed upon FDC in spleens from *Prnp* ^+/-^→*Prnp* ^+/-^ control mice (*P* = 0.106, *n* = 48 FDC networks/group). E) Morphometric analysis confirmed that the magnitude of the PrP^C^ expression co-localized upon the surfaces sympathetic nerves in the spleens of *Prnp* ^flox/-^→CD21-Cre *Prnp* ^flox/-^, CD21-Cre *Prnp* ^flox/-^→ CD21-Cre *Prnp* ^flox/-^ and CD21-Cre *Prnp* ^flox/-^→*Prnp* ^flox/-^ mice was not significantly different when compared to that observed upon sympathetic nerves in spleens from *Prnp* ^+/-^→*Prnp* ^+/-^ control mice (*p* = 0.400, *n* = 48 sympathetic nerves/group). For all panels *n* = 6 mice/group.

Next, mice were created in which *Prnp* expression was specifically ablated in FDC. To do so, CD21-Cre *Prnp* ^-/-^ mice were crossed with mice carrying a “floxed” *Prnp* gene (*Prnp* ^flox/flox^ mice; [31]). In the progeny CD21-Cre *Prnp* ^flox/-^ mice, *Prnp* expression is conditionally ablated in cells expressing Cre recombinase (CD21-expressing FDC and mature B cells). To restrict the *Prnp*-ablation to FDC, CD21-Cre *Prnp* ^flox/-^ mice were lethally γ-irradiated and grafted with bone marrow from Cre-deficient *Prnp* ^flox/-^ mice (*Prnp* ^flox/-^→CD21-Cre *Prnp* ^flox/-^ mice). We also performed bone marrow transfers from CD21-Cre *Prnp* ^flox/-^ donors into CD21-Cre *Prnp* ^flox/-^ recipients (CD21-Cre *Prnp* ^flox/-^ mice→CD21-Cre *Prnp* ^flox/-^ mice), CD21-Cre *Prnp* ^flox/-^ donors into Cre-deficient *Prnp* ^flox/-^ mice (CD21-Cre *Prnp* ^flox/-^→*Prnp* ^flox/-^ mice), and *Prnp* ^+/-^ donors into *Prnp* ^+/-^ recipients (*Prnp* ^+/-^→*Prnp* ^+/-^ mice) as controls (Figure 5A). Spleens, tails and blood from 6 mice from each group were examined 100 days after bone marrow transfusion. PCR analysis of DNA isolated from the spleens, blood and tails of *Prnp* ^flox/-^→CD21-Cre *Prnp* ^flox/-^ mice confirmed that efficient Cre-mediated DNA recombination and *Prnp*-ablation was restricted to the FDC-containing stromal compartment of the spleen (Figure 5B). In *Prnp* ^flox/-^→CD21-Cre *Prnp* ^flox/-^ mice the recombined *Prnp* ^stop/-^ allele (*Prnp* ^deflox^) was detected in the spleen, but not blood and tail. Thus these data indicate that in the spleens of *Prnp* ^stop/-^→CD21-Cre *Prnp* ^stop/-^ mice Cre-mediated recombination and *Prnp*-ablation is restricted to FDC and not B cells.

IHC analysis showed that in the spleens of *Prnp* ^flox/-^→CD21-Cre *Prnp* ^flox/-^ mice and CD21-Cre *Prnp* ^flox/-^ mice→CD21-Cre *Prnp* ^flox/-^ mice FDC did not express PrP^C^ whereas high levels were associated with TH-positive sympathetic nerves (Figure 5C). In the absence of Cre-recombinase expression by FDC in CD21-Cre *Prnp* ^flox/-^→*Prnp* ^flox/-^ mice, high levels of PrP^C^ were expressed by FDC and sympathetic nerves (Figure 5C).

Morphometric analysis confirmed that the magnitude of the PrP^C^ expression co-localized upon the surfaces of FDC in the spleens of *Prnp* ^flox/-^→CD21-Cre *Prnp* ^flox/-^ mice and CD21-Cre *Prnp* ^flox/-^ mice→CD21-Cre *Prnp* ^flox/-^ mice was substantially lower than that observed upon FDC in spleens from *Prnp* ^+/-^→*Prnp* ^+/-^ control mice (*P*<1×10^-24^ and *P*<1×10^−23^, respectively, *n* = 48 FDC/group) and not significantly different when compared to background levels (Figure 5D). In contrast, in the absence of Cre-recombinase expression by FDC in CD21-Cre *Prnp* ^flox/-^→*Prnp* ^flox/-^ mice, PrP^C^ expression was not significantly different from the level observed upon FDC in spleens from *Prnp* ^+/-^→*Prnp* ^+/-^ control mice (*P*<0.106; Figure 5D). In contrast, morphometric analysis showed that the magnitude of the PrP^C^ expression co-localized upon the surfaces sympathetic nerves in the spleens of *Prnp* ^flox/-^→CD21-Cre *Prnp* ^flox/-^, CD21-Cre *Prnp* ^flox/-^→ CD21-Cre *Prnp* ^flox/-^ and CD21-Cre *Prnp* ^flox/-^→*Prnp* ^flox/-^ mice was similar to that observed upon sympathetic nerves in spleens from *Prnp* ^+/-^→*Prnp* ^+/-^ control mice (*p* = 0.400, *n* = 48 sympathetic nerves/group). Together, these data confirm that in the spleens of *Prnp* ^flox/-^→CD21-Cre *Prnp* ^flox/-^ mice the *Prnp* ablation is specifically restricted to FDC.

### Effect of FDC-specific *Prnp*-ablation on FDC status and splenic microarchitecture

**Figure 6:**
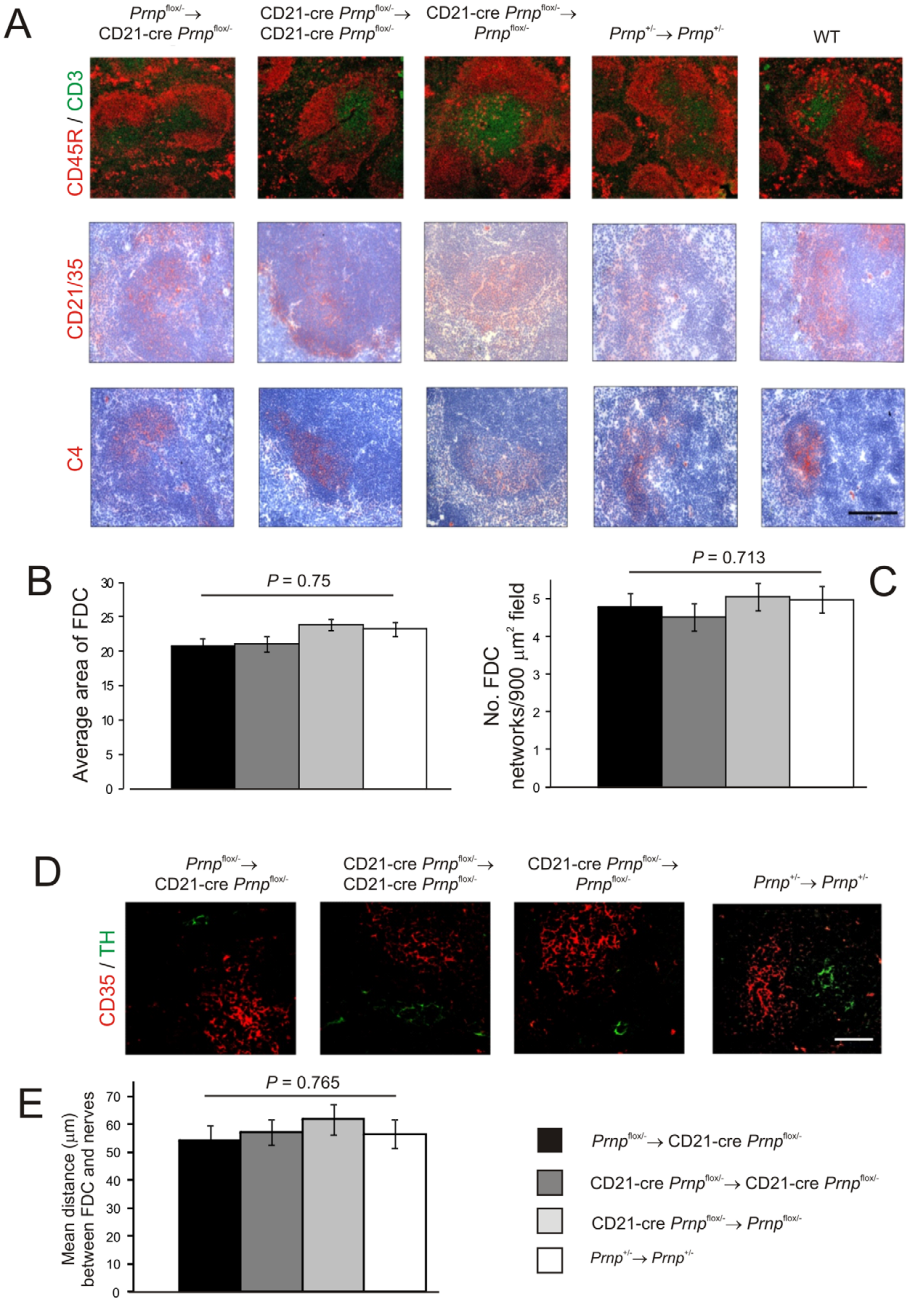
Effect of FDC-restricted PrP^C^-ablation on FDC status. A) IHC analysis of the status of FDC (C4-binding cells and CD21/CD35^+^ cells; red), B cells expressing CD45R (red), and CD3^+^ T cells (green). Morphometric analysis confirmed that there were was no significant difference in the size (B) and number (C) of the CD35^+^ FDC networks in spleens of mice from each mouse group (*n* = 32 FDC networks/group). D and E), Comparison of the sympathetic innervation in spleens from *Prnp* ^flox/-^→CD21-Cre *Prnp* ^flox/-^, CD21-Cre *Prnp* ^flox/-^→CD21-Cre *Prnp* ^flox/-^, CD21-Cre *Prnp* ^flox/-^→*Prnp* ^flox/-^mice and *Prnp* ^+/-^→*Prnp* ^+/-^ control mice. D) IHC detection of TH-positive sympathetic nerves (green) and FDCs (CD35^+^ cells; red). Scale bar, 50 µm. E) Quantitative analysis of the relative positioning of the FDC networks and sympathetic nerves showed there was no significant difference in the average distance between these cell populations in spleens from each mouse group (*P* = 0.765, *n* = 48 FDC networks/group). For all panels *n* = 6 mice/group.

Data in the current study definitively demonstrate that FDC express high levels of PrP^C^ but the role PrP^C^ plays in FDC function and homeostasis is not known. IHC analysis showed that the microarchitecture of the FDC networks from *Prnp*-ablated *Prnp* ^flox/-^→CD21-Cre *Prnp* ^flox/-^ mice were normal when compared to control mice (Figure 6A). Furthermore, no significant difference was observed in the size (*P = 0.*750, *n* = 32) and number (*P = 0.*713, *n* = 32 of the FDC networks in spleens from each mouse group (Figure 6B & C, respectively). The relative positioning of the FDC and sympathetic nerves was likewise similar in spleens from each mouse group (Figure 6D & E; *P*<0.765, *n* = 48).

**Figure 7:**
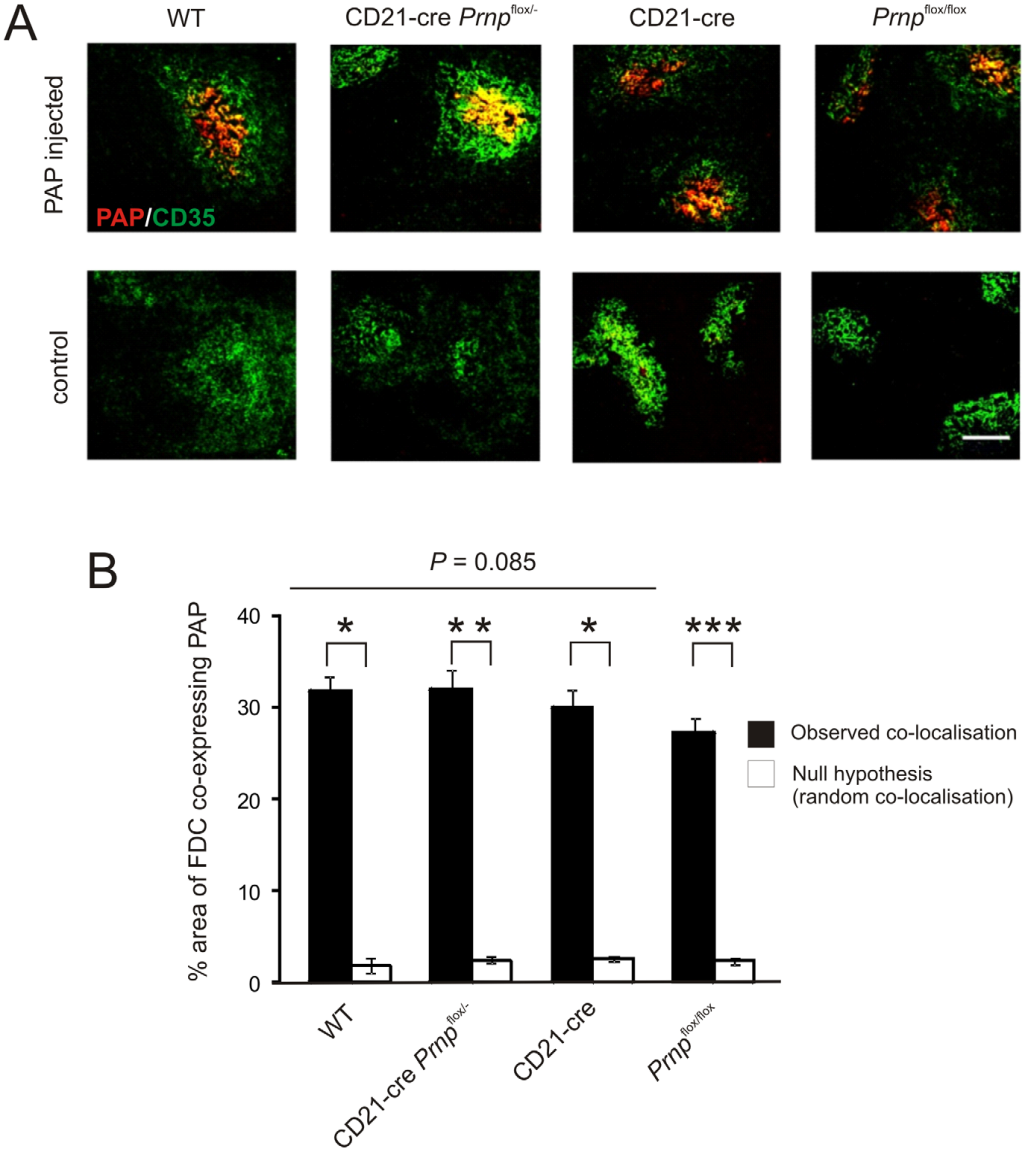
Effect of FDC-restricted PrP^C^-ablation on immune complex trapping. A) Mice were passively immunized with preformed PAP immune complexes, and 24 h later, the presence of immune complexes (red) upon FDC (CD35^+^ cells, green) assessed by IHC. Scale bar, 100 µm. B) Morphometric analysis confirmed that the magnitude of the immune complex-trapping co-localized upon the surfaces of FDC from *Prnp*-ablated *Prnp* ^flox/-^→CD21-Cre *Prnp* ^flox/-^ mice was not significantly different from that observed in spleens from control mice. This analysis also confirmed that the immune complexes were preferentially associated with FDC in these tissues and significantly greater than the null hypothesis that the pixels were randomly distributed. *, *P*<1×10^−21^; **, *P*<1×10^−32^; *** *P*<9×10^−28^; *n* = 40 FDC networks/group. For all panels *n* = 6 mice/group.

FDC characteristically trap and retain native antigen on their surfaces in the form of immune complexes, consisting of antigen-antibody and/or complement components. Antigens trapped on the surface of FDC are considered to promote immunoglobulin-isotype class switching, affinity maturation of naïve IgM^+^ B cells and the maintenance of immunological memory [38]–[42]. Indeed, prions are also considered to be acquired by FDC as complement-opsonized immune complexes [15]–[18]. To determine whether antigen retention by *Prnp*-ablated FDC was affected six mice from each group were passively immunized with preformed PAP immune complexes, and 24 h later, the presence of FDC-associated immune complexes identified by IHC (Figure 7) and the presence of peroxidase activity (data not shown). No significant difference in the magnitude of immune complex trapping could be detected between FDC from *Prnp*-ablated *Prnp* ^flox/-^→CD21-Cre *Prnp* ^flox/-^ mice and control mice (Figure 7; *P = 0.*85, *n* = 40/group). Together, these data demonstrate that *Prnp*-ablation does not impair FDC status or their ability to trap and retain immune complexes.

### FDC-restricted PrP^C^-ablation blocks prion replication in the spleen

**Figure 8:**
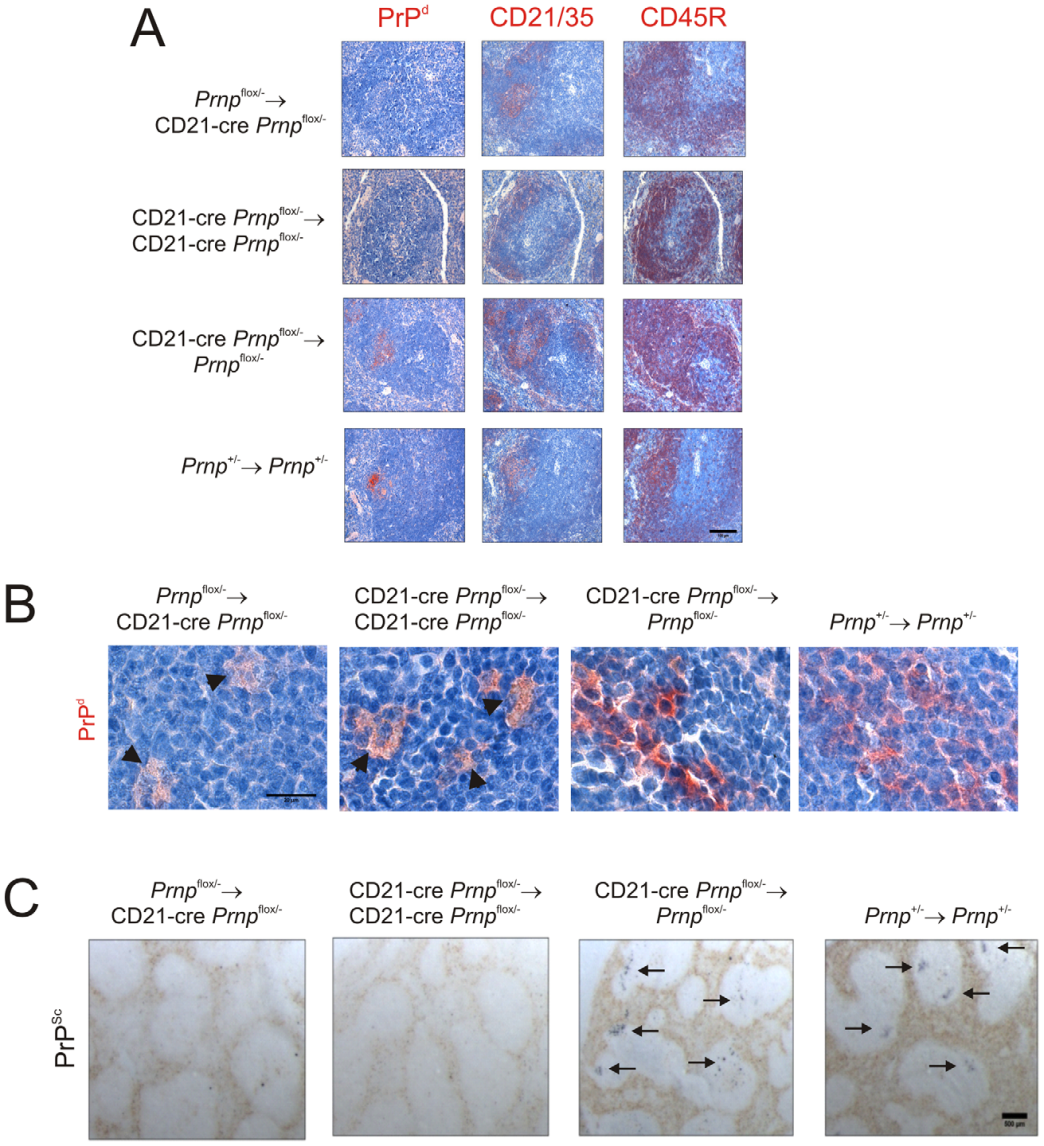
Effect of FDC-restricted PrP^C^-ablation on PrP^Sc^ accumulation in the spleen. Mice were injected i.p. with the ME7 scrapie agent and tissues collected 70 days after exposure. A) High levels of PrP^d^ (red, left-hand column) were detected in association with FDC (red, middle column) in the B cell follicles (red, right-hand column) of spleens from CD21-Cre *Prnp* ^flox/-^→*Prnp* ^flox/-^mice and *Prnp* ^+/-^→*Prnp* ^+/-^ control mice that contained PrP^C^-expressing FDC. B) High magnification images of the sites of PrP^d^ accumulation (red) at 70 days post-injection with scrapie. C) PET blot analysis of analysis of adjacent sections by PET-immunoblot analysis confirmed presence of PK-resistant PrP^Sc^ (blue/black). In contrast, no PrP^Sc^ was detected in spleens of *Prnp* ^flox/-^→CD21-Cre *Prnp* ^flox/-^ and CD21-Cre *Prnp* ^flox/-^→CD21-Cre *Prnp* ^flox/-^ mice that lacked PrP^C^-expressing FDC. In the spleens of some of these mice, low levels of PrP^d^ were occasionally localised within tingible body macrophages (B, arrowheads). A, scale bar  = 100 µm. B, scale bar  = 20 µm. C, scale bar  = 500 µm. Arrows indicate PrP^Sc^ accumulation upon FDC. For all panels *n* = 4 mice/group.

Next, the effect of FDC-specific *Prnp*-ablation on prion replication by FDC was determined. Mice were injected i.p. with ME7 scrapie prions and spleens from 4 mice from each group collected 70 days after exposure. As anticipated, heavy PrP^d^ (Figure 8A and B) and PrP^Sc^ (Figure 8C) accumulations consistent with localisation upon FDC were detected in spleens from control mice (*Prnp* ^+/-^→*Prnp* ^+/-^ mice) and mice in which *Prnp* was ablated only in mature B cells (CD21-Cre *Prnp* ^flox/-^→*Prnp* ^flox/-^ mice). In the spleens in which cellular PrP^C^ was ablated only on FDC (*Prnp* ^flox/-^→CD21-Cre *Prnp* ^flox/-^ mice), or FDC and mature B cells (CD21-Cre *Prnp* ^flox/-^→CD21-Cre *Prnp* ^flox/-^ mice), no PrP accumulations were observed upon FDC (Figure 8A–C). Consistent with data above (Figure 4), in spleens of mice with PrP^C^-deficient FDC PrP accumulations were only occasionally observed within tingible body macrophages (Figure 8A and B, arrowheads; Figure S1). We also analysed prion infectivity levels in spleens from *Prnp* ^flox/-^→CD21-Cre *Prnp* ^flox/-^ mice in which cellular PrP^C^ expression was ablated only on FDC (Figure S2; *n* = 3). Consistent with data above this analysis showed that in the absence of PrP^c^ expression only on FDC the accumulation of high levels of prion infectivity in the spleen was blocked (Figure S2). Taken together, these data show that in the specific absence of PrP^C^ expression FDC are unable to sustain prion replication upon their surfaces and as a consequence the agent is scavenged by tingible body macrophages.

### FDC-restricted PrP^C^-ablation does not influence prion disease and susceptibility when infection is established directly within the CNS

**Figure 9:**
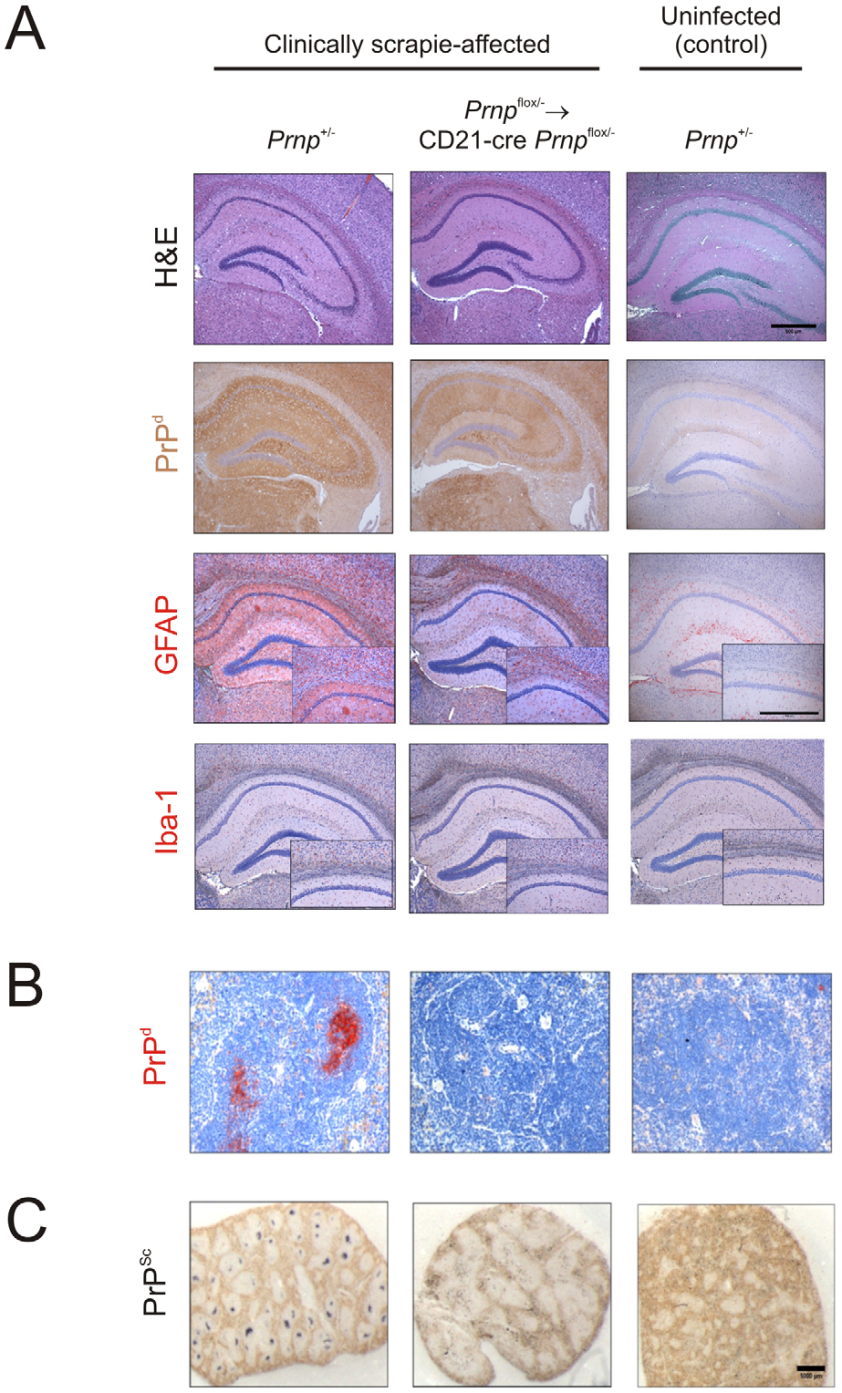
Effect of FDC-restricted PrP^C^-ablation on PrP^Sc^ accumulation in the brains and spleens of scrapie-affected mice. Control mice (*Prnp* ^+/-^ mice) and *Prnp* ^flox/-^→CD21-Cre *Prnp* ^flox/-^ mice that lacked PrP^C^-expressing FDC were injected i.c. with the scrapie agent directly into the CNS. Brains and spleens were collected from clinically scrapie-affected mice to compare the neuropathology and cellular sites of PrP^Sc^ accumulation. A) High levels of spongiform pathology (H&E, upper row), heavy accumulations of PrP^d^ (brown, second row), reactive astrocytes expressing GFAP (brown, third row) and active microglia expressing Iba-1 (brown, bottom row) were detected in the hippocampi of the brains of all clinically scrapie-affected mice. Scale bars, 500 µm. B) High levels of PrP^d^ (red) were detected in association with FDC in spleens from clinically scrapie-affected control mice that contained PrP^C^-expressing FDC. C) PET blot analysis of analysis of adjacent sections by PET-immunoblot analysis confirmed the presence of PK-resistant PrP^Sc^ (blue/black). In contrast, no PrP^d^ or PrP^Sc^ was detected in spleens of *Prnp* ^flox/-^→CD21-Cre *Prnp* ^flox/-^ that lacked PrP^C^-expressing FDC. Scale bars  = 500 µm.

When mice with PrP^C^-ablated FDC (*Prnp* ^flox/-^→CD21-Cre *Prnp* ^flox/-^ mice) were injected intracerebrally (i.c.) with the ME7 scrapie agent strain directly into the CNS all mice succumbed to clinical signs of scrapie approximately 300 days after exposure with incubation periods indistinguishable from those of *Prnp* ^+/-^ control mice [43] (*Prnp* ^flox/-^→CD21-Cre *Prnp* ^flox/-^, 297±4 days, *n* = 4; *Prnp* ^+/-^, 290±4 days, *n* = 5; *P* = 0.386). Histopathological analysis showed that brains from all clinically-affected mice from each group displayed the characteristic spongiform pathology, astrogliosis, microgliosis and PrP^d^ accumulation typically associated with terminal infection with the ME7 scrapie agent (Figure 9A). Following i.c.-injection with the ME7 scrapie agent, high levels of PrP^Sc^ accumulate upon FDC and are maintained for the duration of the incubation period [13](Figure 9B and C). However, FDC are not critical for ME7 scrapie pathogenesis when infection is established directly within the CNS [4], [13], [35], [44]. In the spleens from clinically-scrapie affected mice in which PrP^C^ expression was specifically ablated only on FDC (*Prnp* ^flox/-^→CD21-Cre *Prnp* ^flox/-^ mice), PrP^Sc^ replication upon FDC was also blocked. These data how that FDC do not simply trap and retain prions after their release from infected neurones in the CNS. These data also confirm that the *Prnp*-ablation in *Prnp* ^flox/-^→CD21-Cre *Prnp* ^flox/-^ mice was specific to FDC and had no effect on prion neuropathogenesis and disease susceptibility when the infection was established directly in the CNS.

### Effect of FDC-restricted PrP^C^-ablation prion neuroinvasion after i.p. exposure

Studies in mice show that efficient prion neuroinvasion from peripheral sites of exposure is dependent upon the presence of FDC in lymphoid tissues [1], [4], [35], [44]–[46]. Next, the effect of FDC-specific *Prnp*-ablation on prion neuroinvasion via the peritoneal route was determined. Unfortunately, due to the advanced ages of the mice in this experiment, some succumbed to ageing-related inter-current illness. As there was a 100 days interval between the time of lethal γ-irradiation/bone marrow reconstitution and prion infection, many mice were approximately 500–600 days old when culled. However, most mice with PrP^C^-expressing FDC in their spleens succumbed to clinical prion disease after i.p. injection (*Prnp* ^+/-^→ *Prnp* ^+/-^ control mice, *n* = 5/7; CD21-Cre *Prnp* ^flox/-^→*Prnp* ^flox/-^ mice, *n* = 3/6; Table S1). Histopathological analysis showed that brains from all clinically-affected mice from these groups displayed the characteristic spongiform pathology, astrogliosis, microgliosis and PrP^d^ accumulation typically associated with terminal infection with the ME7 scrapie agent (Figure S3, third and fourth columns). In contrast, none of the mice with PrP^C^-ablated FDC (*Prnp* ^flox/-^→CD21-Cre *Prnp* ^flox/-^ mice, *n* = 0/6; CD21-Cre *Prnp* ^flox/-^→CD21-Cre *Prnp* ^flox/-^ mice, *n* = 0/7) succumbed to clinical prion disease during their life-spans (Table S1). Although we cannot exclude the possibility that if the clinically-negative mice with PrP^C^-ablated FDC mice had lived longer some may have succumbed to clinical prion disease after substantially extended incubation periods, no PrP^d^ or other characteristic histopathological hallmarks of prion disease were detected in their brains (Figure S3, first two columns). Together, these data suggest that in the specific absence of PrP^C^ expression on FDC neuroinvasion following peripheral exposure is impaired.

## Discussion

These data definitively demonstrate that FDC are essential sites of prion replication in lymphoid tissues. In order to precisely establish the role of FDC in prion pathogenesis two unique compound transgenic mouse models were created in which PrP^C^ expression was specifically “switched on” or “off” only on FDC. Our data confirm that FDC express high levels of PrP^C^ and do not simply acquire it from other host cells. Furthermore, we show that following peripheral exposure PrP^C^-expressing FDC alone are sufficient to sustain high levels of prion replication in the spleen. Accordingly, when PrP^C^-expression was specifically ablated only on FDC prion replication in the spleen was blocked. These data likewise demonstrate that FDC do not simply acquire prions after their release from other infected host cells. Our analysis showed that the effects of *Prnp*-ablation on prion replication in the spleen were specific to FDC and had no effect on prion neuropathogenesis when the infection was established directly in the CNS. In the absence of PrP^C^ expression on FDC the PrP^Sc^ from the initial inoculum appeared to be scavenged by tingible body macrophages resident within the B cell follicles. Together, these data definitively demonstrate that FDC are the critical early sites of prion replication in lymphoid tissues. This study is the first to demonstrate that the specific ablation of a cellular protein only on FDC, without apparent consequences for FDC status and function, blocks the replication of an important pathogen in the spleen.

FDC reside in the primary B cell follicles and germinal centres of lymphoid tissues and are a completely distinct cell lineage from bone-marrow-derived classical dendritic cells [47]–[49]. FDC possess many slender and convoluted dendritic processes which provide the FDC with an extremely large surface area. This helps the FDC to efficiently trap and retain large amounts of native antigen in the form of immune complexes, consisting of antigen-antibody and/or complement components. The longevity of FDC ensures that antigen is retained upon their surfaces for long periods [50], [51]. Antigens trapped on the surface of FDC are considered to promote immunoglobulin-isotype class switching, affinity maturation of naïve IgM^+^ B cells and the maintenance of immunological memory [38]–[42]. FDC are also considered to aid the clearance of apoptotic B lymphocytes [52], and play a role in infection with human immunodeficiency virus [53] and the pathogenesis of chronic inflammatory and autoimmune diseases [54] and peripherally-acquired prion infections.

A number of studies have addressed the role of FDC in prion pathogenesis. They show that prion replication in the spleen and subsequent neuroinvasion are both impaired in immunodeficient mice that lack FDC [4], [44], [45], or following their temporary de-differentiation [1], [35], [46]. Although the precise identity of FDC precursor cells is unknown, other studies have exploited their non-haematopoietic-origin to address their role in prion pathogenesis. In these bone marrow chimera studies, mismatches were created in *Prnp* expression between the FDC-containing stromal and haematopoietic compartments by grafting bone marrow cells from PrP-deficient (*Prnp* ^-/-^) mice into PrP-expressing wild-type mice, and *vice versa* [13], [14]. Using this approach FDC and all other stromal cells were derived from the recipient, whereas lymphocytes and other haematopoietic lineages were derived from the donor cells. Following peripheral exposure prion accumulation upon FDC was only detected in the spleens of mice with a *Prnp*-expressing stromal compartment.

While the above studies clearly show that the presence of FDC is important for prion replication in the spleen, it was not possible to dissociate the *Prnp* expression status of FDC from that of the nervous system and all other non-haematopoietic host-cell populations and therefore precisely characterise the role of FDC in prion neuroinvasion [13], [14]. This is important for a number of reasons. Firstly, prion infection can occur within inflammatory PrP^C^-expressing stromal cells that are distinct from FDC [23]. Secondly, the FDC's ability to bind exosomes may have lead to the wrong interpretation to be made in earlier studies describing their ontology [55]. Most evidence indicates that FDC do not derive from haematopoietic precursors [29], [49]. However, the detection of donor bone marrow derived MHC class-I molecules, and other donor-derived antigens, on the surface of FDC in recipient mice was considered evidence of FDC precursor cells within bone marrow [55]. With hindsight these observations are most likely due to the FDC's capacity to acquire exosome-associated antigens from other cell types [21]. Both PrP^C^ and PrP^Sc^ can be released from cells in association with exosomes [20]. The possibility, therefore, cannot be excluded that FDC passively acquire prions after their release in exosomes from other infected non-haematopoietic cell populations. Finally, FDC characteristically trap and retain immune complexes on their surfaces. FDC express negligible levels of complement component C4 at the mRNA level but the detection of abundant activated C4 on their surfaces by IHC using mAb FDC-M2 (as used in this study) is indicative of the capture and retention of immune complexes by FDC [32]. Opsonising complement components and cellular CR are likewise considered to play an important role in the retention of prions by FDC [15], [16], [18]. Thus FDC may simply act as concentrating depots for prion-containing complement-opsonized immune complexes.

The practical hurdles that are encountered when attempting to isolate highly purified FDC from lymphoid tissues have made detailed analysis of their pathobiological functions extremely difficult. The main issues include: contamination with other cell types such as B cells and tingible body macrophages which express MFGE8 (FDC-M1), a common marker used to identify FDC [52], [56], low yield, and their dependence on constitutive lymphotoxin β receptor-stimulation to maintain their differentiated state [57]. FDC and mature B cells express high levels of *Cr2* which encodes the complement receptors CR2/CR1 (CD21/35) [18], [27]. A previous study used CD21-cre mice to study FDC-specific gene function [27]. In the current study, our data confirm that Cre/loxP-mediated DNA recombination was specific to FDC and mature B cells in CD21-cre mice, and could be restricted to FDC by transfusing the mice with Cre-deficient bone marrow. In some Cre transgenic mouse lines Cre-toxicity is encountered whereby Cre recombinase causes mis-recombination, DNA damage and death of *Cre*-expressing cells [30]. However, our analysis suggested no significant effect of Cre-expression on the number, size and status of FDC networks and B cell follicles. CD21-Cre mice are therefore a powerful *in vivo* tool in which to study FDC-specific gene expression and function.

Expression of PrP^C^ is mandatory for host cells to sustain prion infection [43]. In the current study to establish whether FDC actively amplify prions a compound transgenic mouse model was created using the CD21-cre mouse line to specifically “switch on” PrP^C^ expression only on FDC (*Prnp* ^stop/-^→CD21-Cre *Prnp* ^stop/-^ mice). As a consequence, only FDC in these mice had the potential to be actively infected with and replicate prions. Our analysis showed that expression of PrP^C^ only on FDC was sufficient to sustain high levels of PrP^Sc^ accumulation upon FDC in the spleen after peripheral prion exposure. These data definitively demonstrate that FDC are the critical sites of prion replication in lymphoid tissues. Ultrastructural analysis of the cellular compartments within which PrP^d^ localizes upon/within FDC has failed to show any intracellular accumulation. Instead the PrP^d^ appears to be restricted to the plasmalemma of their dendritic processes [58]. This implies that early *de novo* PrP^Sc^ conversion occurs upon the surface of FDC.

A second compound transgenic mouse model was created in which PrP^C^ expression was specifically “switched off” only on FDC (*Prnp* ^flox/-^→CD21-Cre *Prnp* ^flox/-^ mice). If, as shown above, FDC do actively amplify prions, then one would also expect the specific ablation of PrP^C^ expression only on FDC to block prion replication in the spleen. Our data confirmed this to be the case. As PrP^C^ expression in all other host cells (eg: neurones) in these mice was unaffected, these data clearly show that FDC do not simply acquire prions following release from other infected host cells, even in mice with clinical prion disease in the brain. IHC analysis implied that in the spleens of mice with PrP-deficient FDC the prions appeared to be scavenged by tingible body macrophages resident within the B cell follicles. The lack of detection of PrP^d^ within tingible body macrophages in the spleens of clinically-affected mice with PrP-deficient FDC (Figure 9) clearly demonstrates that these cells are not alternative sites of replication of ME7 scrapie prions. High levels of prions rapidly accumulate within the spleen and other lymphoid tissues within weeks of peripheral exposure. The magnitude of the prion accumulation within the spleen rapidly reaches a plateau level which is maintained for the duration of the disease [13], [44]. The maintenance of this plateau may be the consequence of a competitive state whereby FDC act to amplify prions above the threshold required to achieve neuroinvasion, whereas phagocytic cells such as macrophages act to destroy them [59], [60]. Indeed increased numbers of PrP^d^-containing tingible body macrophages are found within the B cell follicles of TSE-affected animals [58]. Thus, our data suggest that in the specific absence of PrP^C^ expression by FDC the initial inoculum is phagocytosed and gradually degraded by mononuclear phagocytes such as tingible body macrophages [59], [60]. These data are congruent with data from our earlier study which likewise occasionally detected trace levels of prions from the initial inoculum within tingible body macrophages in the spleens of mice with a PrP^C^-deficient FDC-containing stromal compartment [13].

The density of sympathetic nerves can significantly influence the amount of prion accumulation in the spleen [33]. In the current study the distribution of TH-positive sympathetic nerves in the spleens of the FDC-specific gene targeted mouse lines was not adversely affected. Furthermore, when prions were injected directly to the brain, FDC-specific *Prnp* ablation had no influence on the onset of clinical disease or the neuropathology. These data provide strong evidence that the effects of Cre-mediated *Prnp* ablation on prion replication in the spleen were specific to FDC and not due to unregulated ablation of PrP^C^ expression within the nervous system. In the current study PrP^Sc^ accumulation upon PrP^C^-ablated FDC (*Prnp* ^flox/-^→CD21-Cre *Prnp* ^flox/-^ mice) was blocked even in spleens from i.c. injected clinically-scrapie affected mice. These data contrast those reported by Crozet and colleagues [61] which used Tg(OvPrP4) mice that express the ovine *PRNP* gene under the control of the neuron-specific enolase promoter on a murine *Prnp* ^-/-^ background. As a consequence ovine PrP^C^ is expressed only in neurones. In contrast to data in the current study, when Tg(OvPrP4) mice were injected i.c. with a high dose of natural sheep scrapie PrP^Sc^ was detected in the germinal centres of their spleens. The reasons for this discrepancy are uncertain. However, the expression of PrP^C^ in the neuronal compartment of Tg(OvPrP4) mice is 2-4X higher than in controls. In the current study in mice in which PrP^C^ was ablated only on FDC (*Prnp* ^flox/-^→CD21-Cre *Prnp* ^flox/-^ mice) the expression of murine *Prnp* in Cre-deficient cells such as neurones is controlled by the endogenous *Prnp* promoter and expressed at similar levels to controls (Figure 5E). In the presence of increased PrP^C^ expression on neurones it is plausible that greater prion replication occurred within the peripheral nervous system, which may have been subsequently trapped on the surface of the FDC and scavenged by macrophages as the prion burden increased. Similarly, hyper-innervation of the spleen likewise leads to increase prion burden in this tissue [33].

In conclusion, our data demonstrate that PrP^C^-expressing FDC are the essential sites of prion replication in lymphoid tissues. Indeed, PrP^C^-expression on FDC alone was sufficient to sustain high levels of prion replication. In contrast, the specific ablation of PrP^C^ expression on FDC blocked prion replication. Although FDC have the capacity to bind exosomes and immune complexes which may contain PrP^Sc^, this finding clearly demonstrates that FDC do not simply passively acquire prions from other infected cell populations such as neurones. Previous data show treatments which impair the status or immune complex-trapping function of FDC reduce prion susceptibility after peripheral exposure [1], [16], [35], [46], [62]. The demonstration that *Prnp*-ablation only on FDC blocked splenic prion replication without apparent consequences for FDC status represents a novel opportunity to prevent neuroinvasion by modulation of PrP^C^ expression on FDC.

## Materials and Methods

### Ethics statement

All studies using experimental mice and regulatory licences were approved by both The Roslin Institute's and University of Edinburgh's Protocols and Ethics Committees. All animal experiments were carried out under the authority of a UK Home Office Project Licence within the terms and conditions of the strict regulations of the UK Home Office ‘Animals (scientific procedures) Act 1986'. Where necessary, anaesthesia appropriate for the procedure was administered, and all efforts were made to minimize harm and suffering. Mice were humanely culled using by a UK Home Office Schedule One method.

### Mice

The CD21-Cre [26], ROSA26^flox/flox^ reporter strain [28], *Prnp* ^-/-^ [12] mice and tga20 mice over-expressing PrP^c^ [63] were generated as described previously. *Prnp* ^flox/flox^ mice have *loxP* sites flanking exon 3 of the *Prnp* gene [31]. *Prnp* ^stop/-^ mice have a floxed β-geo cassette inserted into intron 2 of the *Prnp* gene upstream of exon 3 [31]. Mice were maintained under SPF conditions.

### Genotype confirmation by PCR analysis

Prior to their use in experiments, the genotype of each mouse was confirmed by PCR analysis. DNA was prepared from tails, blood and spleens using the DNeasy blood and tissue kit (Qiagen, Crawley, UK) according to the manufacturer's instructions. Where indicated DNA samples were analysed for presence of *Cre*, *LacZ*, *Prnp* ^+/+^, *Prnp* ^-/-^, *Prnp* ^flox^, recombined *Prnp* ^flox^ (*Prnp^de-^* ^flox^), *Prnp* ^stop^ and recombined *Prnp* ^stop^ (*Prnp* ^stop(R)^) using the primers listed in Table 1. PCR products were resolved by electrophoresis through a 1.0% agarose gel containing 0.002% GelRed (Biotium, Cambridge Biosciences Ltd, Cambridge, UK).

**Table 1:** PCR primers used to confirm the genotypes of mice used in this study.

Allele	Details	Primer sequence	Product size/s (bp)
*Cre*	Fwd	CGAGTGATGAGGTTCGCAAGAACC	786
	Rev	GCTAAGTGCCTTCTCTACACCTGC	300
*LacZ*	Fwd	TACCACAGCGGATGGTTCGG	300
	Rev	GTGGTGGTTATGCCGATCGC	
*Prnp^flox^*	1	AATGGTTAAACTTTCGTTAAGGAT	*Prnp^de-flox^* 344
	2	GCCGACATCAGTCCACATAG	*Prnp^flox^* 210
	3	GGTTGACGCCATGACTTTC	*Prnp^WT^* 167
*Prnp^null^*	Fwd	GCCATCACGAGATTTCGATT	1,200
	Rev	ATCCCACGATCAGGAAGATG	
*Prnp^WT^*	Fwd	TCATCCCACGATCAGGAAGATGAG	600
	Rev	ATGGCGAACCTTGGCTACTGGCTG	
*Prnp^stop^*	1	ACAAATGTGGTATGGCTGATTATG	*Prnp^stop^* 1,000
	2	ATGATGATTGAACAAGATGGATTG	*Prnp^stop(R)^* 840
	3	TACCACGAAGTCCGGGATAG	*Prnp^WT^* 750
	4	GGCAGAGGCTAAGGACAACA	

Fwd, forward primer; Rev, reverse primer; (R), Cre-mediated DNA recombined allele.

### γ-Irradiation and bone-marrow reconstitution

Bone-marrow from the femurs and tibias of donor mice was prepared as single-cell suspensions (3×10^7^–4×10^7^ viable cells/ml) in HBSS (Invitrogen, Paisley, UK). Recipient adult (6–8 weeks old) mice were γ-irradiated (950 rad) and 24 h later reconstituted with 100 µl bone-marrow by injection into the tail vein. Recipient mice were used in subsequent experiments as described 100 days after bone marrow reconstitution to allow sufficient time for removal of long-lived B lymphocyte populations and their replacement from the donor bone marrow.

### Histological assessment of *Lacz* expression

Tissues were first immersed in *LacZ* fixative [PBS (pH 7.4) containing 2% paraformaldehyde, 0.2% gluteraldehyde, 0.02% Nonidet P40, 0.01% sodium deoxycholate, 5 mM EGTA, 2 mM MgCl_2_] and washed in *LacZ* wash buffer [PBS (pH 7.4) containing 0.02% Nonidet P40, 0.01% sodium deoxycholate, 2 mM MgCL_2_]. Tissues were subsequently incubated in 15% (wt/vol) sucrose in PBS overnight followed by a further overnight incubation in 30% (wt/vol) sucrose in PBS and embedded in Tissue-Tek O.C.T. compound (Bayer PLC, Newbury, UK). Serial sections (thickness 8 µm) were cut on cryostat and stained overnight at 37°C with *LacZ* staining solution (Glycosynth, Warrington, UK). Staining reaction was stopped by washing in *LacZ* wash buffer followed by dH_2_O. Sections were counterstained with nuclear fast red (Vector Laboratories, Peterborough, UK).

### Prion exposure and disease monitoring

For i.c. or i.p. exposure mice were injected with 20 µl of a 1% (v/w) scrapie brain homogenate prepared from mice terminally-affected with ME7 scrapie prions (containing approximately 1×10^4^ i.c. ID_50_ units). Following exposure, mice were coded and assessed blindly for signs of clinical disease and culled at a standard clinical endpoint [64]. Survival times were recorded for mice that did not develop clinical signs of disease and were culled when they showed signs of intercurrent disease. Scrapie diagnosis was confirmed blindly on coded sections by histopathological assessment of vacuolation in the brain. For the construction of lesion profiles, vacuolar changes were scored in nine grey-matter areas of brain as described [65]. Where indicated, some four mice from each group were culled at the times indicated post injection with scrapie and tissues taken for further analysis. For bioassay of scrapie agent infectivity, individual half spleens were prepared as 10% (wt/vol) homogenates in physiological saline. Groups of four tga20 indicator mice were injected i.c. with 20 µl of each homogenate. The scrapie titre in each sample was determined from the mean incubation period in the indicator mice, by reference to a dose/incubation period response curve for ME7 scrapie-infected spleen tissue serially titrated in tga20 mice using the relationship: *y* = 9.4533–0.0595*x* (*y*, = log ID_50_ U/20 µl of homogenate; *x*, incubation period; R^2^ = 0.9562). As the expression level of cellular PrP^c^ controls the prion disease incubation period, tga20 mice overexpressing PrP^c^ are extremely useful as indicator mice in prion infectivity bioassays as they succumb to disease with much shorter incubation times than conventional mouse strains [63].

### IHC and immunofluorescent analyses

Spleens were removed and snap-frozen at the temperature of liquid nitrogen. Serial frozen sections (10 µm in thickness) were cut on a cryostat and immunostained with the following antibodies: FDCs were visualized by staining with mAb 7G6 to detect CR2/CR1 (CD21/CD35; BD Biosciences PharMingen), mAb FDC-M2 to detect C4 (AMS Biotechnology, Oxon, UK) or mAb 8C12 to detect CR1 (CD35; BD Biosciences PharMingen). Cellular PrP^C^ was detected using PrP-specific polyclonal antibody (pAb) 1B3 [66]. B cells were detected using mAb B220 to detect CD45R (Caltag, Towcester, UK), or anti-CD19 (BD biosciences PharMingen). Marginal zone B cells were detected using mAb 1B1 to detect CD1d (BD Biosciences PharMingen). Sympathetic nerves were detected using tyrosine hydroxylase (TH)-specific pAb (Chemicon Europe).

For the detection of disease-specific PrP (PrP^d^) in spleens and brains, tissues were fixed in periodate-lysine-paraformaldehyde fixative and embedded in paraffin wax. Sections (thickness, 6 µm) were deparaffinised, and pre-treated to enhance the detection of PrP^d^ by hydrated autoclaving (15 min, 121°C, hydration) and subsequent immersion formic acid (98%) for 5 min [67]. Sections were then immunostained with 1B3 PrP-specific pAb. For the detection of EGF-like module-containing mucin-like hormone receptor-like 1 (EMR1)-expressing macrophages, paraffin-embedded spleen sections were micro-waved in citric acid buffer (pH 6.0) for 10 min. Endogenous peroxidase activity was blocked using 1% hydrogen peroxidase in methanol, and macrophages detected using rat mAb F4/80 to detect EMR1 (clone CI:A3-1, AbD Serotec). For the detection of astrocytes, brain sections were immunostained with anti-glial fibrillary acidic protein (GFAP; DAKO, Ely, UK). For the detection of microglia, deparaffinised brain sections were first pre-treated with Target Retrieval Solution (DAKO) and subsequently immunostained with anti-ionized calcium-binding adaptor molecule 1 (Iba-1; Wako Chemicals GmbH, Neuss, Germany). Immunolabelling was revealed using HRP-conjugated to the avidin-biotin complex (Novared kit, Vector laboratories, Peterborough, UK). Paraffin-embedded tissue (PET) immunoblot analysis was used to confirm the PrP^d^ detected by immunohistochemistry was proteinase K (PK)-resistant PrP^Sc^ [34]. Membranes were subsequently immunostained with 1B3 PrP-specific pAb.

For light microscopy, following the addition of primary antibodies, biotin-conjugated species-specific secondary antibodies (Stratech, Soham, UK) were applied followed by alkaline phosphatase or HRP coupled to the avidin/biotin complex (Vector Laboratories). Vector Red (Vector Laboratories) and diaminobenzidine (DAB; Sigma Aldrich, Dorset, UK) were used as substrates, respectively, and sections were counterstained with haematoxylin to distinguish cell nuclei. For fluorescent microscopy, following the addition of primary antibody, species-specific secondary antibodies coupled to Alexa Fluor 488 (green), Alexa Fluor 594 (red) dyes or Alexa Fluor 647 (blue) dyes (Invitrogen, Paisley, UK) were used. Sections were mounted in fluorescent mounting medium (DakoCytomation) and examined using a Zeiss LSM5 confocal microscope (Zeiss, Welwyn Garden City, UK).

### Image analysis

Digital microscopy images were analyzed using ImageJ software (http://rsbweb.nih.gov/ij/) as described [68]. Intensity thresholds were first applied and then the number of pixels of each colour (black, red, green, yellow) were then automatically counted and presented as a proportion of the total number of pixels in each area under analysis. The preferential co-localisation of fluorochromes was determined by comparisons of the observed distribution of colours with those predicted by the null hypothesis that each element of positive staining was randomly and independently distributed. Values found to be significantly greater than the null hypothesis confirm significant co-localisation of fluorochromes. Spleens from 6 mice from each group were analyzed. From each spleen, 2 sections were studied and on each section data from 4 individual FDC networks collected. Thus, for each mouse group data from a total of 48 individual FDC were analysed. Similarly, data from 48 images from each group were analyzed to determine the preferential co-localisation of fluorochromes upon TH-positive sympathetic nerves within the spleen. A one-way ANOVA test was then used to compare the null hypothesis (that the pixels were randomly distributed) to the observed levels of co-localisation.

### Passive immunization

To assess antigen trapping by FDC *in vivo*, mice were passively immunized by intravenous injection with 100 µl preformed PAP immune complexes (Sigma). Spleens were removed 24 h later and the presence of FDC-associated immune complexes identified by IHC.

### Statistical analyses

Data are presented as mean ± SE. Unless indicated otherwise, significant differences between samples in different groups were sought by one-way ANOVA. Values of *P*<0.05 were accepted as significant.

## Supporting Information

Figure S1 **In the absence of PrP^C^ expression by follicular dendritic cells prions are scavenged by tingible body macrophages in the spleen.** Mice were injected i.p. with ME7 scrapie prions. Spleens from CD21-Cre *Prnp* ^stop/-^→*Prnp* ^stop/-^ mice (in which cellular PrP^C^ was expressed only on B cells, upper row), *Prnp* ^flox/-^→CD21-Cre *Prnp* ^flox/-^ mice (with FDC-restricted PrP^C^ ablation, middle row) and CD21-Cre *Prnp* ^flox/-^→CD21-Cre *Prnp* ^flox/-^ mice (in which PrP^C^ expression was ablated on FDC and B cells, lower row) were collected 70 days after i.p. infection. Due to the absence of PrP^C^-expressing FDC prion replication in these tissues was blocked. However, in the spleens of some of these mice, low levels of PrP^d^ (left-hand column, red) were occasionally localised within cells with characteristics typical of tingible body macrophages. These cells contained the remnants of many phagocytosed apoptotic lymphocytes (*tingible bodies*, arrowheads) and expressed the tissue macrophage marker EGF-like module-containing mucin-like hormone receptor-like 1 (EMR1) detected by mAb F4/80 (right-hand column, brown). Data are representative of spleens from at least 4 mice from each group. Sections are counterstained with haematoxylin (blue). Scale bar, 20 µm.
(PDF)

Figure S2 **Follicular dendritic cell-specific PrP^c^ expression alone is sufficient to sustain high levels of prion infectivity in the spleen.** Prion infectivity levels were assayed spleens from control mice (*Prnp* ^+/-^→*Prnp* ^flox/-^ mice), *Prnp* ^stop/-^→CD21-Cre *Prnp* ^stop/-^ mice in which cellular PrP^C^ was expressed only on FDC and *Prnp* ^flox/-^→CD21-Cre *Prnp* ^flox/-^ mice with FDC-restricted PrP^C^ ablation (*n* = 3/group) collected 70 days after i.p. with ME7 scrapie prions. Prion infectivity titres were determined by transmission of tissue homogenates into groups of 4 indicator tga20 mice. Each point represents data derived from an individual spleen. Data below the horizontal line indicate disease incidence in the recipient mice <100% and considered to contain trace levels of prion infectivity. High levels of prion infectivity were detected in spleens of control mice and those in which cellular PrP^C^ was expressed only on FDC (left-hand and middle panels, respectively). However, this accumulation was blocked in spleens with FDC-restricted PrP^C^ ablation as only trace levels of infectivity were detected (right-hand panel).
(PDF)

Figure S3 **Effect of FDC-restricted PrP^C^-ablation on disease pathogenesis within the brain after i.p. prion exposure.** Mice were injected i.p. with ME7 scrapie prions. Brains were collected from clinically scrapie-affected mice and mice which were free of the clinical signs of prion disease at the time of cull and the neuropathology within each brain compared. High levels of spongiform pathology (H&E, upper row), heavy accumulations of PrP^d^ (brown, second row), reactive astrocytes expressing GFAP (brown, third row) and active microglia expressing Iba-1 (brown, bottom row) were detected in the hippocampi of the brains of all clinically scrapie-affected control mice (right-hand column, *n* = 5) and mice in which PrP^C^ expression was ablated in B cells only (CD21-Cre *Prnp* ^flox/-^→*Prnp* ^flox/-^ mice, third column, *n* = 3). In contrast, none of the mice with PrP^C^-ablated FDC (FDC-restricted, *Prnp* ^flox/-^→CD21-Cre *Prnp* ^flox/-^ mice, first column, *n* = 6; FDC and B cells, CD21-Cre *Prnp* ^flox/-^→CD21-Cre *Prnp* ^flox/-^ mice, second column, *n* = 7) developed clinical signs of prion disease during their life-spans or histopathological signs of prion disease in their brains. Scale bar, = 500 µm. Clin., presence of clinical signs of scrapie at the time of cull; Path., histopathological detection of spongiform pathology in the brain; dpi, days post i.p. prion infection.
(PDF)

Table S1 **Effect of FDC-restricted** ***Prnp*** **ablation on prion disease pathogenesis after i.p. exposure.**
(DOCX)
