# Supplementary material for: Follicular Dendritic Cell-Specific Prion Protein (PrPc) Expression Alone Is Sufficient to Sustain Prion Infection in the Spleen
Source: PLoS Pathog. 2011 Dec 1;7(12):e1002402. doi: 10.1371/journal.ppat.1002402 (PMC3228802; doi:10.1371/journal.ppat.1002402)
Supplement: Figure S1 — In the absence of PrPC expression by follicular dendritic cells prions are scavenged by tingible body macrophages in the spleen. Mice were injected i.p. with ME7 scrapie prions. Spleens from CD21-Cre Prnp stop/-→Prnp stop/- mice (in which cellular PrPC was expressed only on B cells, upper row), Prnp flox/-→CD21-Cre Prnp flox/- mice (with FDC-restricted PrPC ablation, middle row) and CD21-Cre Prnp flox/-→CD21-Cre Prnp flox/- mice (in which PrPC expression was ablated on FDC and B cells, lower row) were collected 70 days after i.p. infection. Due to the absence of PrPC-expressing FDC prion replication in these tissues was blocked. However, in the spleens of some of these mice, low levels of PrPd (left-hand column, red) were occasionally localised within cells with characteristics typical of tingible body macrophages. These cells contained the remnants of many phagocytosed apoptotic lymphocytes (tingible bodies, arrowheads) and expressed the tissue macrophage marker EGF-like module-containing mucin-like hormone receptor-like 1 (EMR1) detected by mAb F4/80 (right-hand column, brown). Data are representative of spleens from at least 4 mice from each group. Sections are counterstained with haematoxylin (blue). Scale bar, 20 µm. (PDF) [file ppat.1002402.s001.pdf]

Follicular Dendritic Cell-Specific Prion Protein (PrP^c) Expression Alone is Sufficient to Sustain Prion Infection in the Spleen

Running head: Role of Follicular Dendritic Cells in Prion Pathogenesis

Laura McCulloch, Karen L. Brown, Barry Bradford, John Hopkins, Mick Bailey, Klaus Rajewsky, Jean C. Manson & Neil A. Mabbott

Figure S1. In the absence of PrP^C expression by follicular dendritic cells prions are scavenged by tingible body macrophages in the spleen

Mice were injected i.p. with ME7 scrapie prions. Spleens from CD21-Cre *Prnp*$^{stop/-}$ →*Prnp*$^{stop/-}$ mice (in which cellular PrP^C was expressed only on B cells, upper row), *Prnp*$^{flox/-}$→CD21-Cre *Prnp*$^{flox/-}$ mice (with FDC-restricted PrP^C ablation, middle row) and CD21-Cre *Prnp*$^{flox/-}$→CD21-Cre *Prnp*$^{flox/-}$ mice (in which PrP^C expression was ablated on FDC and B cells, lower row) were collected 70 days after i.p. infection. Due to the absence of PrP^C-expressing FDC prion replication in these tissues was blocked. However, in the spleens of some of these mice, low levels of PrP^d (left-hand column, red) were occasionally localised within cells with characteristics typical of tingible body macrophages. These cells contained the remnants of many phagocytosed apoptotic lymphocytes (*tingible bodies*, arrowheads) and expressed the tissue macrophage marker EGF-like module-containing mucin-like hormone receptor-like 1 (EMR1) detected by mAb F4/80 (right-hand column, brown). Data are representative of spleens from at least 4 mice from each group. Sections are counterstained with haematoxylin (blue). Scale bar, 20 μm.

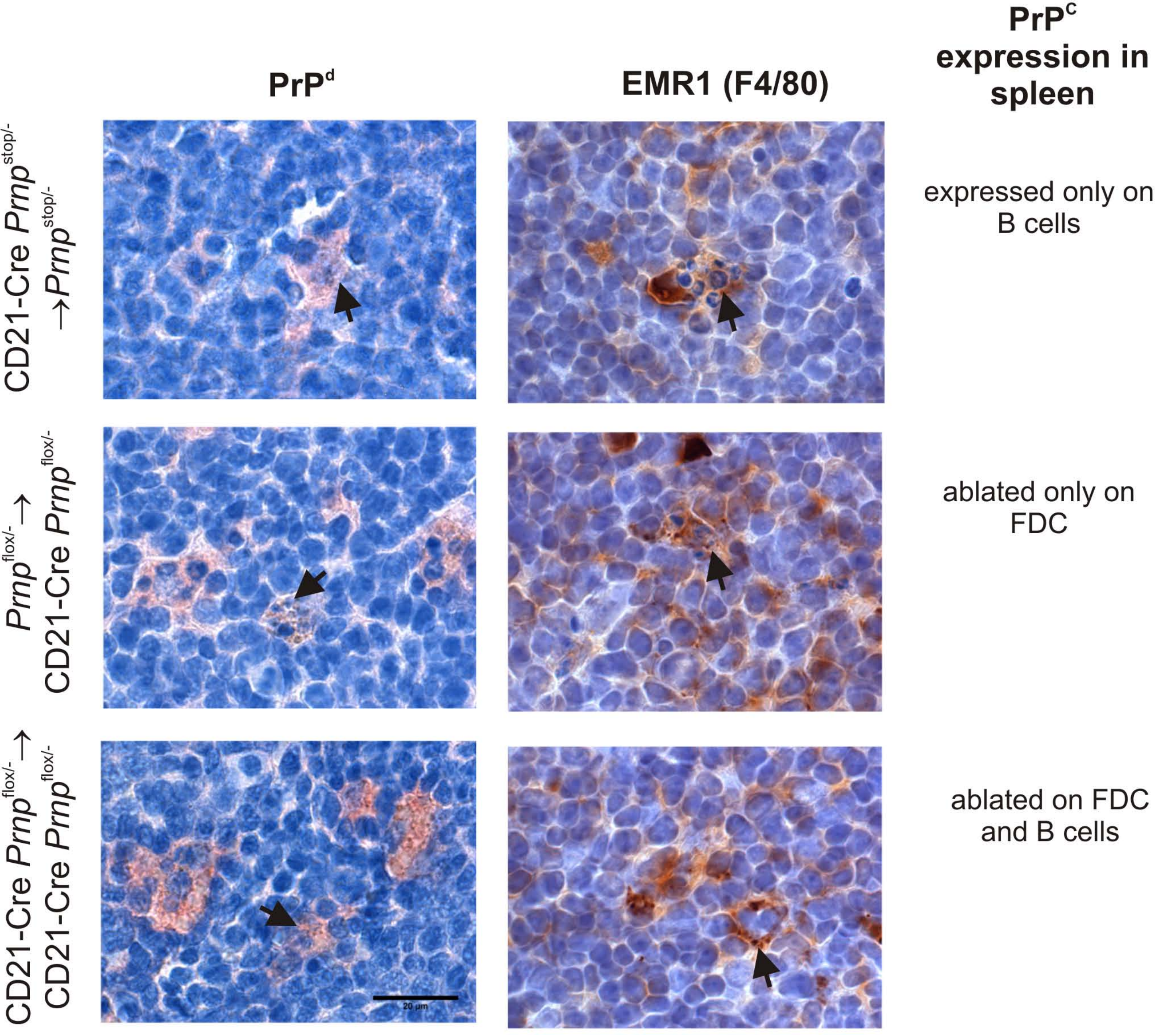
PrP^d
EMR1 (F4/80)
PrP^c expression in spleen
CD21-Cre Prnp^stop/- →Prnp^stop/-
expressed only on B cells
Prnp^flox/- → CD21-Cre Prnp^flox/-
ablated only on FDC
CD21-Cre Prnp^flox/- → CD21-Cre Prnp^flox/-
ablated on FDC and B cells
20 µm
