# Supplementary material for: Follicular Dendritic Cell-Specific Prion Protein (PrPc) Expression Alone Is Sufficient to Sustain Prion Infection in the Spleen
Source: PLoS Pathog. 2011 Dec 1;7(12):e1002402. doi: 10.1371/journal.ppat.1002402 (PMC3228802; doi:10.1371/journal.ppat.1002402)
Supplement: Figure S2 — Follicular dendritic cell-specific PrPc expression alone is sufficient to sustain high levels of prion infectivity in the spleen. Prion infectivity levels were assayed spleens from control mice (Prnp +/-→Prnp flox/- mice), Prnp stop/-→CD21-Cre Prnp stop/- mice in which cellular PrPC was expressed only on FDC and Prnp flox/-→CD21-Cre Prnp flox/- mice with FDC-restricted PrPC ablation (n = 3/group) collected 70 days after i.p. with ME7 scrapie prions. Prion infectivity titres were determined by transmission of tissue homogenates into groups of 4 indicator tga20 mice. Each point represents data derived from an individual spleen. Data below the horizontal line indicate disease incidence in the recipient mice <100% and considered to contain trace levels of prion infectivity. High levels of prion infectivity were detected in spleens of control mice and those in which cellular PrPC was expressed only on FDC (left-hand and middle panels, respectively). However, this accumulation was blocked in spleens with FDC-restricted PrPC ablation as only trace levels of infectivity were detected (right-hand panel). (PDF) [file ppat.1002402.s002.pdf]

Follicular Dendritic Cell-Specific Prion Protein (PrP^c) Expression Alone is Sufficient to Sustain Prion Infection in the Spleen

Running head: Role of Follicular Dendritic Cells in Prion Pathogenesis

Laura McCulloch, Karen L. Brown, Barry Bradford, John Hopkins, Mick Bailey, Klaus Rajewsky, Jean C. Manson & Neil A. Mabbott

Figure S2. Follicular dendritic cell-specific PrP^c expression alone is sufficient to sustain high levels of prion infectivity in the spleen

Prion infectivity levels were assayed spleens from control mice (*Prnp*$^{+/-}$→*Prnp*$^{flox/-}$ mice), *Prnp*$^{stop/-}$→CD21-Cre *Prnp*$^{stop/-}$ mice in which cellular PrP^C was expressed only on FDC and *Prnp*$^{flox/-}$→CD21-Cre *Prnp*$^{flox/-}$ mice with FDC-restricted PrP^C ablation (*n* = 3/group) collected 70 days after i.p. with ME7 scrapie prions. Prion infectivity titres were determined by transmission of tissue homogenates into groups of 4 indicator tga20 mice. Each point represents data derived from an individual spleen. Data below the horizontal line indicate disease incidence in the recipient mice <100% and considered to contain trace levels of prion infectivity. High levels of prion infectivity were detected in spleens of control mice and those in which cellular PrP^C was expressed only on FDC (left-hand and middle panels, respectively). However, this accumulation was blocked in spleens with FDC-restricted PrP^C ablation as only trace levels of infectivity were detected (right-hand panel).

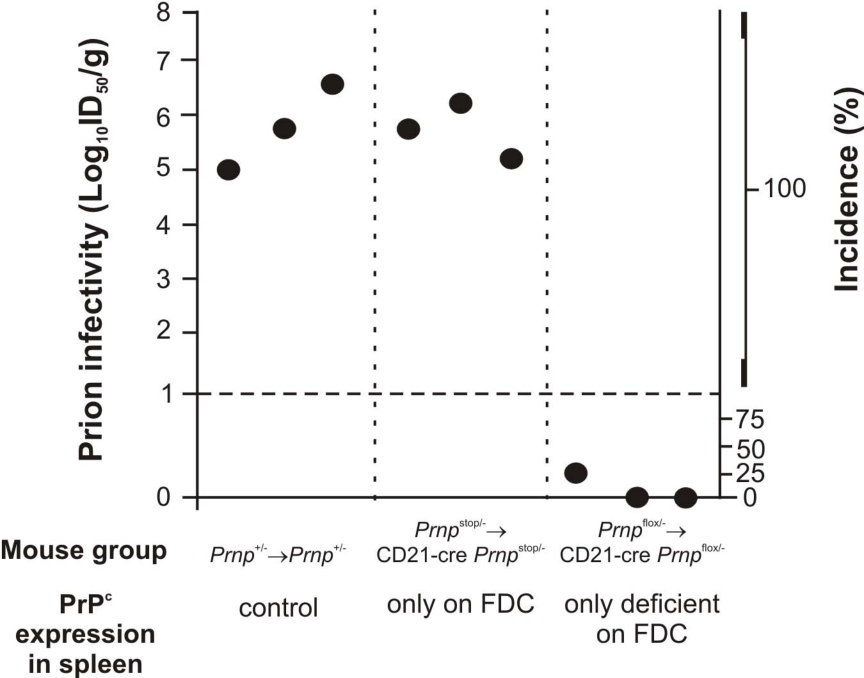
Prion infectivity (Log10ID50/g)
8
7
6
5
4
3
2
1
0
Incidence (%)
100
75
50
25
0
Mouse group
Prnp+/-→Prnp+/-
Prnpstop/-→
CD21-cre Prnpstop/-
Prnpflox/-→
CD21-cre Prnpflox/-
PrPc
expression
in spleen
control
only on FDC
only deficient
on FDC
