# Supplementary material for: Follicular Dendritic Cell-Specific Prion Protein (PrPc) Expression Alone Is Sufficient to Sustain Prion Infection in the Spleen
Source: PLoS Pathog. 2011 Dec 1;7(12):e1002402. doi: 10.1371/journal.ppat.1002402 (PMC3228802; doi:10.1371/journal.ppat.1002402)

Follicular Dendritic Cell-Specific Prion Protein (Prp^c) Expression Alone is Sufficient to Sustain Prion Infection in the Spleen

Running head: Role of Follicular Dendritic Cells in Prion Pathogenesis

Laura McCulloch, Karen L. Brown, Barry Bradford, John Hopkins, Mick Bailey, Klaus Rajewsky, Jean C. Manson & Neil A. Mabbott

Figure S3. Effect of FDC-restricted PrP^C-ablation on disease pathogenesis within the brain after i.p. prion exposure.

Mice were injected i.p. with ME7 scrapie prions. Brains were collected from clinically scrapie-affected mice and mice which were free of the clinical signs of prion disease at the time of cull and the neuropathology within each brain compared. High levels of spongiform pathology (H&E, upper row), heavy accumulations of PrP^d (brown, second row), reactive astrocytes expressing GFAP (brown, third row) and active microglia expressing Iba-1 (brown, bottom row) were detected in the hippocampi of the brains of all clinically scrapie-affected control mice (right-hand column, *n* = 5) and mice in which PrP^C expression was ablated in B cells only (CD21-Cre $Prnp^{flox/-}$ →$Prnp^{flox/-}$ mice, third column, *n* = 3). In contrast, none of the mice with PrP^C-ablated FDC (FDC-restricted, $Prnp^{flox/-}$→CD21-Cre $Prnp^{flox/-}$ mice, first column, *n* = 6; FDC and B cells, CD21-Cre $Prnp^{flox/-}$→CD21-Cre $Prnp^{flox/-}$ mice, second column, *n* = 7) developed clinical signs of prion disease during their life-spans or histopathological signs of prion disease in their brains. Scale bar, = 500 μm. Clin., presence of clinical signs of scrapie at the time of cull; Path., histopathological detection of spongiform pathology in the brain; dpi, days post i.p. prion infection.

Figure S3. McCulloch *et al.*

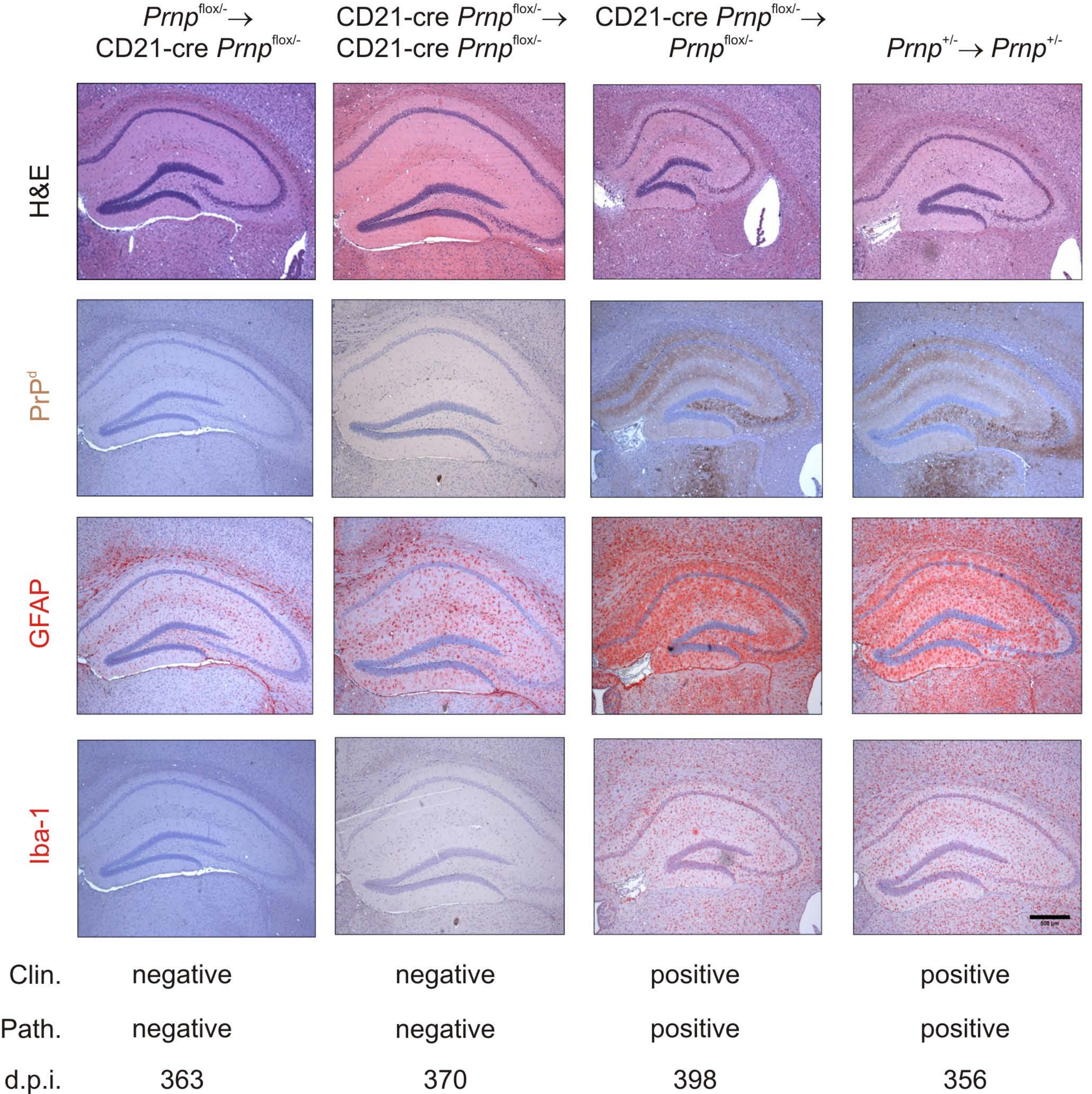

Supplement: Figure S3 — Effect of FDC-restricted PrPC-ablation on disease pathogenesis within the brain after i.p. prion exposure. Mice were injected i.p. with ME7 scrapie prions. Brains were collected from clinically scrapie-affected mice and mice which were free of the clinical signs of prion disease at the time of cull and the neuropathology within each brain compared. High levels of spongiform pathology (H&E, upper row), heavy accumulations of PrPd (brown, second row), reactive astrocytes expressing GFAP (brown, third row) and active microglia expressing Iba-1 (brown, bottom row) were detected in the hippocampi of the brains of all clinically scrapie-affected control mice (right-hand column, n = 5) and mice in which PrPC expression was ablated in B cells only (CD21-Cre Prnp flox/-→Prnp flox/- mice, third column, n = 3). In contrast, none of the mice with PrPC-ablated FDC (FDC-restricted, Prnp flox/-→CD21-Cre Prnp flox/- mice, first column, n = 6; FDC and B cells, CD21-Cre Prnp flox/-→CD21-Cre Prnp flox/- mice, second column, n = 7) developed clinical signs of prion disease during their life-spans or histopathological signs of prion disease in their brains. Scale bar, = 500 µm. Clin., presence of clinical signs of scrapie at the time of cull; Path., histopathological detection of spongiform pathology in the brain; dpi, days post i.p. prion infection. (PDF) [file ppat.1002402.s003.pdf]
