# Supplementary material for: Follicular Dendritic Cell-Specific Prion Protein (PrPc) Expression Alone Is Sufficient to Sustain Prion Infection in the Spleen
Source: PLoS Pathog. 2011 Dec 1;7(12):e1002402. doi: 10.1371/journal.ppat.1002402 (PMC3228802; doi:10.1371/journal.ppat.1002402)
Supplement: Table S1 — Effect of FDC-restricted Prnp ablation on prion disease pathogenesis after i.p. exposure. (DOCX) [file ppat.1002402.s004.docx]

**Follicular Dendritic Cell-Specific Prion Protein (PrP^c^) Expression Alone is Sufficient to Sustain Prion Infection in the Spleen**

Running head: Role of Follicular Dendritic Cells in Prion Pathogenesis

**Laura McCulloch, Karen L. Brown, Barry Bradford, John Hopkins, Mick Bailey, Klaus Rajewsky, Jean C. Manson & Neil A. Mabbott**

Table S1

Table S1: Effect of FDC-restricted *Prnp* ablation on prion disease pathogenesis after i.p. exposure.

| Mouse model^a^ | | Cellular site of *Prnp* ablation in the spleen | | Disease incubation periods or *survival times* (days) | | | Clinical  disease^b^ | | Histopathological  signs of prion disease in the brain^c^ |
| --- | --- | --- | --- | --- | --- | --- | --- | --- | --- |
|  | |  | | |  | |  | |  |
| *Prnp*^flox/-^→  CD21-Cre *Prnp*^flox/-^ | | FDC | | | *307, 326, 335,*  *363*, *377*, *419* | | 0/6 | | 0/6 |
| CD21-Cre *Prnp*^flox/-^→CD21-Cre *Prnp*^flox/-^ | | FDC &  B cells | | | *273, 293, 311, 311, 348, 370, 391* | | 0/7 | | 0/7 |
| CD21-Cre *Prnp*^flox/-^→*Prnp*^flox/-^ | | B cells | | | 286, 398, 440  *245, 326, 326* | | 3/3  0/3 | | 3/3  0/3 |
| *Prnp*^+/-^→*Prnp*^+/-^ | | Control | | | 356, 356, 398,  431, 440 | | 5/5 | | 5/5 |
|  | |  | | | *267, 356* | | 0/2 | | 0/2 |
|  |  | |  | | |  | |  | |

^a^ Mice were lethally γ-irradiated and 24 h later grafted with donor bone marrow as indicated. 100 days after bone marrow transfusion recipient mice were injected i.p. with 20 μl of a 1% (v/w) brain homogenate prepared from mice terminally-affected with ME7 scrapie prions (containing approximately 1 X 10^4^ i.c. ID_50_ units).

^b^ Incidence = no. animals displaying clinical signs of prion disease/no. animals tested.

^c^ Incidence = no. animals with histopathological signs of prion disease in the brain (vacuolation in the neuropil and PrP^Sc^ accumulation)/no. animals tested.
